# The clock components *Period2*, *Cryptochrome1a*, and *Cryptochrome2a* function in establishing light-dependent behavioral rhythms and/or total activity levels in zebrafish

**DOI:** 10.1038/s41598-018-37879-8

**Published:** 2019-01-17

**Authors:** Jun Hirayama, Yikelamu Alifu, Rin Hamabe, Sho Yamaguchi, Jun Tomita, Yusuke Maruyama, Yoichi Asaoka, Ken-ichi Nakahama, Teruya Tamaru, Ken Takamatsu, Nobuhiko Takamatsu, Atsuhiko Hattori, Sachiko Nishina, Noriyuki Azuma, Atsuo Kawahara, Kazuhiko Kume, Hiroshi Nishina

**Affiliations:** 1 Department of Developmental and Regenerative Biology, Medical Research Institute, Tokyo Medical and Dental University (TMDU), Tokyo, Japan; 2 Department of Clinical Engineering, Faculty of Health Sciences, Komatsu University, Ishikawa, Japan; 3 Department of Neuropharmacology, Graduate School of Pharmaceutical Sciences, Nagoya City University, Nagoya, Japan; 4 Department of Biology, College of Liberal Arts and Sciences, Tokyo Medical and Dental University (TMDU), Ichikawa, Japan; 5 Department of Microbiology and Immunology, Yamaguchi University Graduate School of Medicine, Ube, Japan; 6 Department of Cellular Physiological Chemistry, Tokyo Medical and Dental University (TMDU), Tokyo, Japan; 7 Department of Physiology and Advanced Research Center for Medical Science, Toho University School of Medicine, Tokyo, Japan; 8 Department of Biosciences, School of Science, Kitasato University, Sagamihara, Japan; 9 Department of Ophthalmology and Laboratory for Visual Science, National Center for Child Health and Development, Tokyo, Japan; 10 Laboratory for Developmental Biology, Center for Medical Education and Sciences, Graduate School of Medical Science, University of Yamanashi, Yamanashi, Japan

## Abstract

The circadian clock generates behavioral rhythms to maximize an organism’s physiological efficiency. Light induces the formation of these rhythms by synchronizing cellular clocks. In zebrafish, the circadian clock components Period2 (zPER2) and Cryptochrome1a (zCRY1a) are light-inducible, however their physiological functions are unclear. Here, we investigated the roles of zPER2 and zCRY1a in regulating locomotor activity and behavioral rhythms. *zPer2*/*zCry1a* double knockout (DKO) zebrafish displayed defects in total locomotor activity and in forming behavioral rhythms when briefly exposed to light for 3-h. Exposing DKO zebrafish to 12-h light improved behavioral rhythm formation, but not total activity. Our data suggest that the light-inducible circadian clock regulator zCRY2a supports rhythmicity in DKO animals exposed to 12-h light. Single cell imaging analysis revealed that zPER2, zCRY1a, and zCRY2a function in synchronizing cellular clocks. Furthermore, microarray analysis of DKO zebrafish showed aberrant expression of genes involved regulating cellular metabolism, including ATP production. Overall, our results suggest that zPER2, zCRY1a and zCRY2a help to synchronize cellular clocks in a light-dependent manner, thus contributing to behavioral rhythm formation in zebrafish. Further, zPER2 and zCRY1a regulate total physical activity, likely via regulating cellular energy metabolism. Therefore, these circadian clock components regulate the rhythmicity and amount of locomotor behavior.

## Introduction

Diurnal animals display increased locomotor activity during the day and reduced locomotor activity at night^1^. The total amount of locomotor behavior (i.e., activity) is inversely related to the amount of sleep or sleep-like (resting) behavior. Mechanisms associated with energy homeostasis are proposed to regulate the amount an animal sleeps^1–3^, but the molecular mechanisms are not fully understood.

The circadian clock regulates sleep and resting behavior in organisms ranging from bacteria to humans, generating daily physiological rhythms that have been proposed to control the timing of sleep and locomotor activity, but not their total amount^4–7^. Under natural conditions, the circadian clock is entrained to a 24-h day by environmental time cues, with light being the most important^8,9^. The circadian clock is established by cell-autonomous oscillators called cellular clocks^10,11^, which are present in every cell of a living organism. The synchronization of cellular clocks in tissues and organs by environmental signals such as light is required to orchestrate the circadian clock at the organismal level.

In vertebrate cellular clocks, CLOCK or NPAS2 heterodimerizes with BMAL to form a transcription activation complex that facilitates expression of the *Period* (*Per*) and *Cryptochrome* (*Cry*) genes^5,10^. PER and CRY proteins repress CLOCK (NPAS2):BMAL-mediated transcription, establishing the rhythmic gene expression that drives the circadian clock. The CLOCK(NPAS2):BMAL complex also stimulates the expression of clock-controlled genes (Ccgs) within several physiological pathways. One of the Ccgs involved in sleep timing control is *Aanat2*, which encodes a key synthase of the sleep-inducing hormone melatonin^12,13^.

The zebrafish (*Danio rerio*) is an attractive diurnal animal model for the study of vertebrate circadian clocks^14,15^. In particular, the formation of behavioral rhythms in zebrafish is a useful experimental system to analyze light-dependent regulation of the circadian clock *in vivo*. During zebrafish development, organogenesis is completed within two days post fertilization (dpf)^16^. Zebrafish larvae hatch within 4 dpf and then start to display locomotor behavior. Zebrafish cellular clocks are autonomously set in motion during development within 1–4 dpf, but are out of phase with each other in tissues and organs^17,18^. Light synchronizes the phases of the cellular clocks to establish behavioral rhythms^19^.

Zebrafish cultured cells also provide a valuable tool for studying the light-dependent regulation of cellular clocks. In zebrafish cultured cells, light directly resets asynchronous cellular clocks to a common phase by suppressing their transcriptional activities^20–22^. This *in vitro* system facilitates studies on the photic responses of clock genes encoding cellular clock regulators and has revealed several cellular signaling pathways involved in the light response of cellular clocks^23–27^.

We and others have identified acute light-inducible clock genes, namely zebrafish *Period2* (*zPer2*) and *Cryptochrome1a* (*zCry1a*)^28–31^. However, the physiological roles of zCRY1a and zPER2 *in vivo* have not been fully determined. To address these questions, we engineered *zCry1a* and *zPer2* knockout (KO) zebrafish. We found that zPER2 and zCRY1a synchronize cellular clocks in a light-dependent manner to form the behavioral rhythms of zebrafish, and identified zCRY2a as a third light-induced cellular clock synchronizer that contributes to behavioral rhythm formation in zebrafish. In addition, we uncovered unforeseen roles for zPER2 and zCRY1a in regulating the total level of locomotor activity.

## Results

### *zCry1a*^−/−^*zPer2*^−/−^ zebrafish have reduced locomotor activity and defective formation of light-dependent behavioral rhythms

**Figure 1:**
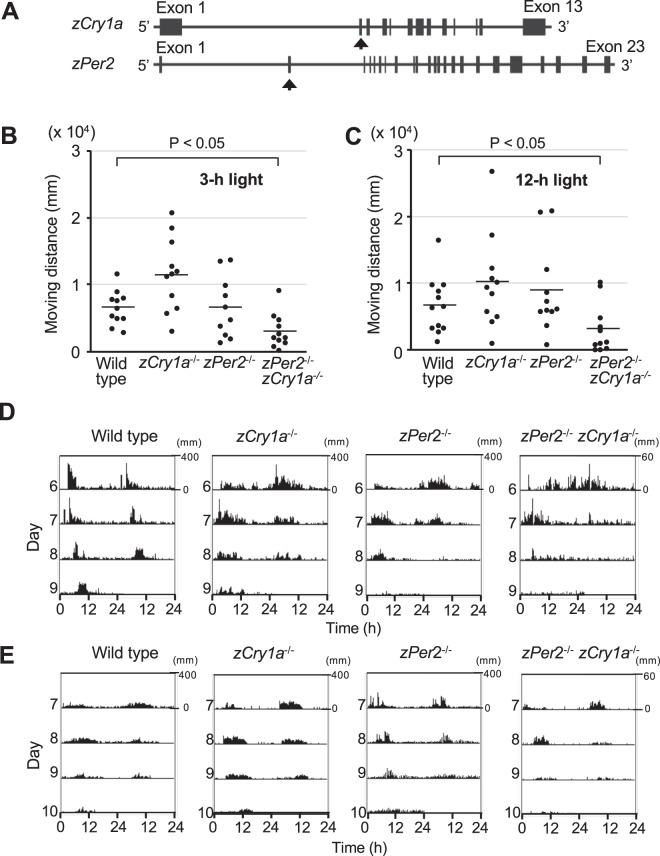
Behavior analyses of *zCry1a* KO, *zPer2* KO, and DKO zebrafish. (**A**) Schematic representation of the genomic structures of the *zCry1a* and *zPer2* genes. Exon regions are shown as solid boxes. Arrows indicate the exons where nonsense mutations were introduced. (**B**) Total locomotor activities of WT, *zCry1a* KO, *zPer2* KO, and DKO zebrafish were calculated from day 6 to day 8. Zebrafish were raised in constant dark (DD) conditions for 5 days after fertilization, and then a 3 h light pulse was administered at the beginning of 6 day after fertilization. Their moving distance was then analyzed in DD condition for 4 days. P < 0.05 (Student’s t-test). [WT (n = 11), *zCry1a* KO (n = 11), *zPer2* KO (n = 10), DKO (n = 11)]. (**C**) Total locomotor activities of WT, *zCry1a* KO, *zPer2* KO, and DKO zebrafish were calculated from day 7 to day 9 after fertilization. Zebrafish larvae were raised in DD conditions for 5 days after fertilization, and then a 12 h light pulse was administered at the beginning of 6 dpf. Their moving distance was then analyzed in DD condition from 7 to 10 days after fertilization. P < 0.05 (Student’s t-test). [WT (n = 12), *zCry1a* KO (n = 11), *zPer2* KO (n = 11), DKO (n = 11)] (**D**) Representative double-plotted activity records (actograms) of WT, *zCry1a* KO, *zPer2* KO, and DKO zebrafish analyzed in B. Note that the range of Y axis of the actogram for DKO animal is distinct from those for WT, *zCry1a* KO, and *zPer2* KO zebrafish. (**E**) Representative double-plotted activity records (actograms) of WT, *zCry1a* KO, *zPer2* KO, and DKO zebrafish analyzed in C. Note that the range of Y axis of the actogram for DKO animal is distinct from those for WT, *zCry1a* KO, and *zPer2* KO zebrafish.

To investigate the physiological roles of zCRY1a and zPER2, we generated *zCry1a*^−/−^ (*zCry1a* KO) and *zPer2*^−/−^ (*zPer2* KO) zebrafish using TALEN technology (Fig. 1A, Supplementary Fig. S1). *zCry1a* KO and *zPer2* KO animals were then crossed to produce *zCry1a*^−/−^ *zPer2*^−/−^ double KO (DKO) zebrafish. It has been reported that exposing 6 dpf zebrafish to light for 3-h or more establishes behavioral rhythms^19,32^. We first evaluated the total locomotor activity of wild type (WT) and KO zebrafish exposed to a single pulse of 3- or 12-h of light (Fig. 1B,C). Interestingly, the DKO zebrafish exposed to 3- or 12-h light showed significantly reduced activity compared to WT animals. This reduced activity was not observed with the single *zPer2* KO or *zCry1a* KO zebrafish.

Next, we analyzed the light-dependent establishment of behavioral rhythms in the KO animals. Behavioral rhythms of locomotor activity were efficiently established in WT, *zCry1a* KO, and *zPer2* KO zebrafish exposed to light for 3-h, but not in WT animals kept in a constant dark (DD) condition (Fig. 1D, Table 1, Supplementary Fig. S2). However, behavioral rhythmicity was reduced in DKO zebrafish exposed to 3-h light. We then evaluated zebrafish exposed to 12-h light (Fig. 1E, Table 1). Behavioral rhythmicity improved in the DKO zebrafish exposed to 12-h compared to 3-h light, although the efficiency of behavior rhythm formation was still lower than in WT animals. Taken together, our results suggest that zCRY1a and zPER2 together play key roles in regulating locomotor activity and light-dependent formation of behavioral rhythms in zebrafish. In addition, they indicate that additional factor(s) besides zPER2 and zCRY1a help to establish normal behavioral rhythms.

**Table 1:** Efficiency of zebrafish’s behavior rhythm formation.

	WT	*zCry1a* ^*−/−*^	*zPer2* ^*−/−*^	*zPer2* ^*−/−*^ *zCry1a* ^*−/−*^
Ratio of rhythmic animals exposed to 3 h light (%)	81	90	80	27
Ratio of rhythmic animals exposed to 12 h light (%)	83	81	81	63

### 12-h light, but not 3-h light, induces expression of *zCry*2a and *zCry2b*

**Figure 2:**
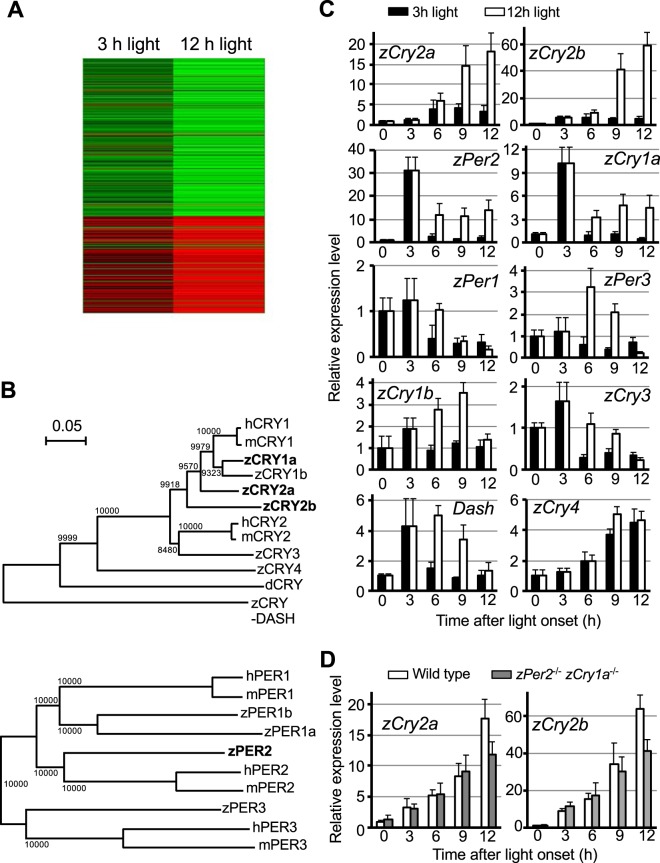
Identification of candidate factor(s) recovering behavioral rhythm of DKO zebrafish exposed to 12 h light. (**A**) Heatmap analysis of microarray data showing hierarchical clustering of differentially expressed genes in DKO zebrafish kept in darkness vs. exposed to light for 3-h (left) and 12-h (right). DKO zebrafish kept in DD conditions for 5 dpf were exposed to light for 3-h or 12-h or kept in darkness, and subjected to sample preparation at 12-h after light onset. Red or green colors indicate differentially up- or downregulated genes, respectively. (**B**) Phylogenetic tree comparing CRY (upper panel) or PER (lower panel) proteins from *Drosophila* (d) zebrafish (z), mouse (m), and human (h). The number at each node indicates the bootstrap probability (%). Scale bar, 0.05 substitutions per site. (**C**) A time course of clock gene expression in zebrafish exposed to light for 3-h (black bar) or 12-h (white bar), determined by RT-PCR. Zebrafish were raised in the darkness until the beginning of 6 dpf. RT-PCR of the indicated genes was examined at the indicated time points after the onset of light (Supplementary Fig. S3). The value at time point 0 was set as 1 for each gene. Values are mean ± S.E.M. of three independent experiments. (**D**) A time course of expression of *zCry2a* and *zCry2b* in WT (white bar) and DKO (gray bar) zebrafish exposed to light for 12 h was examined as in (**C**).

To identify the factor(s) responsible for recovering the behavioral rhythms in the DKO zebrafish exposed to 12-h light, we performed microarray analysis. We compared gene expression levels of the 3- or 12-h light-treated DKO zebrafish, after normalizing to DKO animals kept in darkness (Fig. 2A). We conducted a cluster analysis to identify genes in DKO zebrafish whose expression levels were strongly changed by exposure to 12-h light, but not to 3-h light. We identified two clusters: one cluster contained genes whose expression levels were strongly inhibited by 12-h light but only weakly inhibited by 3-h light, and another cluster contained genes whose expression levels were strongly induced by 12-h light but only weakly induced by 3-h light. The two clock genes *zCry2a* and *zCry2b*, as well as *zCry1a* and *zPer2*, were among the 40 genes most strongly induced in DKO animal exposed to 12-h light (Supplementary Table S1). *zCry2a* and *zCry2b* are evolutionarily related to *zCry1a* (Fig. 2B), and we previously reported that zCRY2a and zCRY2b transcriptionally repress the CLOCK(NPAS2):BMAL complex^33^. Hence, *zCry2a* and *zCry2b* represent candidate factors responsible for recovering the light-dependent behavioral rhythms of DKO zebrafish.

Next, we used RT-PCR to examine the light-dependent expression profiles of *Cry* and *Per* genes in WT zebrafish (Supplementary Fig. S3, Fig. 2C). A 12-h pulse of light induced efficient expression of *zCry2a* and *zCry2b*, reaching high levels at 12-h after light onset, consistent with a previous report^34^. However, a 3-h pulse of light did not induce efficient expression of *zCry2a* and *zCry2b*. In addition, *zCry2a* and *zCry2b* displayed different light induction profiles than *zCry1a* and *zPer2*. Both *zCry1a* and *zPer2* were strongly induced by a pulse of 3- or 12-h light, and maximum expression levels were observed at 3-h after light onset. It should be noted that *zCry2a*, *zCry2b*, *zPer2* and *zCry1a* were not induced in animals kept in the DD condition (Supplementary Fig. S4), verifying their light-dependent expression. Other *Cry* and *Per* gene family members showed substantially lower light-induced expression levels compared to *zCry2a* and *zCry2b* (Fig. 2B,C). Finally, we found that a 12-h light exposure also induced the expression of *zCry2a* and *zCry2b* genes in DKO zebrafish (Fig. 2D). Taken together, our results suggest that zCRY2a and/or zCRY2b might function in establishing the behavioral rhythms of DKO zebrafish exposed to 12-h light.

### Genetic inhibition of *zCry2a* in DKO zebrafish blocks light-dependent formation of behavioral rhythms

**Figure 3:**
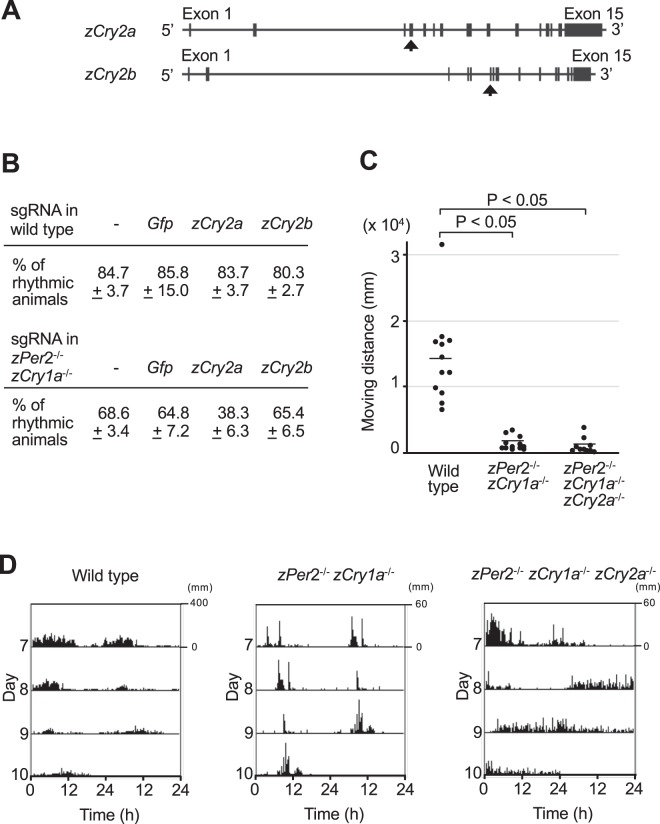
Evaluation of zCRY2a’s role in the light-dependent establishment of zebrafish behavioral rhythms. (**A**) Schematic representation of the genomic structures of *zCry2a* and *zCry2b* genes. Exon regions are shown as solid boxes. Arrows indicate the exons where nonsense mutations were introduced. (**B**) WT or DKO embryos were injected with *Cas9* mRNA and sgRNAs targeting *Gfp*, *zCry2a*, or *zCry2b* genes, and then kept in DD to generate larvae. The zebrafish were exposed to light for 12-h at the beginning of 6 dpf, and their locomotor behavior was then analyzed in DD condition from 7 to 10 dpf. Existence of circadian rhythmicity in behavior was evaluated by ActogramJ software, and the ratio (%) of rhythmic animals for each genotype is shown. Values are mean ± S.E.M. of three independent experiments. [Non-injected WT animal (n = 6, n = 10, and n = 8 for each experiment 1, 2, and 3 respectively); WT animals injected with sgRNA of *Gfp* (n = 10, n = 10, and n = 9 for experiment 1, 2, and 3 respectively), *zCry2a* (n = 6, n = 10, and n = 8 for experiment 1, 2, and 3 respectively), or *zCry2b* (n = 6, n = 9, and n = 10 for experiment 1, 2, and 3 respectively); Non-injected DKO animal (n = 6, n = 11, and n = 8 for experiment 1, 2, and 3 respectively), DKO animals injected with sgRNA of *Gfp* (n = 12, n = 11, and n = 8 for experiment 1, 2, and 3 respectively), *zCry2a* (n = 6, n = 11, and n = 8 for experiment 1, 2, and 3 respectively), or *zCry2b* (n = 5, n = 11, and n = 8 for experiment 1, 2, and 3 respectively)] (**C**) Total locomotor activity of WT, DKO, and TKO zebrafish exposed to 12-h light was calculated from day 7 to day 9. Zebrafish were raised in DD conditions until the beginning of 6 dpf, when a 12-h light pulse was administered. Their locomotor activity was analyzed in DD condition from 7 to 10 dpf. *P < 0.05 (Student’s t-test). [WT (n = 12), DKO KO (n = 12), TKO (n = 10)] (**D**) Representative double-plotted activity records (actograms) of WT, DKO, and TKO zebrafish analyzed in C. The range of Y axis of the actogram for WT animal is distinct from those for DKO and TKO zebrafish.

To test whether zCRY2a and/or zCRY2b are involved in forming light-dependent behavioral rhythms in DKO zebrafish, we used the CRISPR-Cas9 system to inhibit *zCry2a* or *zCry2b* (Fig. 3A, Supplementary Fig. S5A,B). WT or DKO embryos were injected with Cas9 mRNA and single guide RNAs (sgRNAs) targeting the *zCry2a* or *zCry2b* genes, and kept in constant darkness (DD) until they developed into larvae. We exposed the injected zebrafish to light for 12-h at 6 dpf, and analyzed the proportion of animals that displayed behavioral rhythms in DD condition. Inhibition of *zCry2a*, but not *zCry2b*, markedly reduced the ability of DKO animals to form behavioral rhythms (Fig. 3B). In contrast, inhibition of *zCry2a* or *zCry2b* in WT zebrafish had no effect on behavioral rhythm formation. Thus, zCRY2a contributes to the formation of behavioral rhythms in DKO zebrafish exposed to 12-h light.

To extend our findings, we generated *zCry1a*^−/−^ *zPer2*^−/−^ *zCry2a*^−/−^ triple KO (TKO) zebrafish (Figs 1A and 3A, Supplementary Fig. S5C,D). We evaluated total locomotor activity of WT, DKO, and TKO zebrafish exposed to light for 12-h. Both DKO and TKO zebrafish showed significantly lower locomotor activity than WT animals (Fig. 3C). We analyzed their behavior data at high temporal resolution (1-sec bin); because the properties of cumulative probability distribution of continuous immobile period length (rest bout) of WT zebrafish changes around 60 seconds (dashed line in Supplementary Fig. S6), we defined sleep-like resting behavior as a period of 60 or more seconds without movement. We found that the reduced activities in DKO and TKO zebrafish reflected an increase in this resting behavior (Supplementary Fig. S6). This definition of resting behavior in zebrafish is consistent with that reported by another group^35,36^. Based on this definition, we analyzed in detail the resting behavior of DKO and TKO zebrafish, and found that the total length of resting time and the total number of resting periods both increased, and that the mean moving distance during one active period as well as the mean length were decreased compared to WT animals (Table 2). Temporal properties of active and rest behaviors were calculated using the 1-sec bin locomotor activity data, according to the definition of rest as 60 seconds or longer continuous immobility, as described in Materials and Methods.

**Table 2:** Behavior analyses of WT, DKO and TKO animals exposed to 12-h light.

	WT	zPer2^−/−^ zCry1a^−/−^	zPer2^−/−^ zCry1a^−/−^ zCry2a^−/−^
Amount of resting time (h)	7.8 ± 2.0	20.5 ± 1.9	20.6 ± 2.8
Number of active-rest bouts	110.3 ± 49.8	177.1 ± 39.4	171.7 ± 35.8
Mean moving distance during one active period (mm)	178.5 ± 160.6	8.5 ± 6.4	5.7 ± 5.4
Mean length of time of one active period (h)	7.8 ± 2.3	1.1 ± 0.5	1.0 ± 0.7
Ratio of rhythmic animals (%)	91	66	30

Temporal properties of active and rest behaviors were calculated using the 1-sec bin locomotor activity data according to the definition of rest as 60 sec or longer continuous immobility as described in Materials and Methods.

Next, we evaluated the capacity of TKO zebrafish to establish behavioral rhythms (Fig. 3D and Table 2). Notably, behavioral rhythm formation of zebrafish exposed to 12-h light was substantially reduced in TKO animals compared to DKO animals. Taken together, our results reveal that zCRY2a contributes to the establishment of behavioral rhythms in zebrafish exposed to 12-h light.

### Deregulation of cellular clocks causes the defect in light-dependent behavioral rhythm formation in DKO and TKO zebrafish

**Figure 4:**
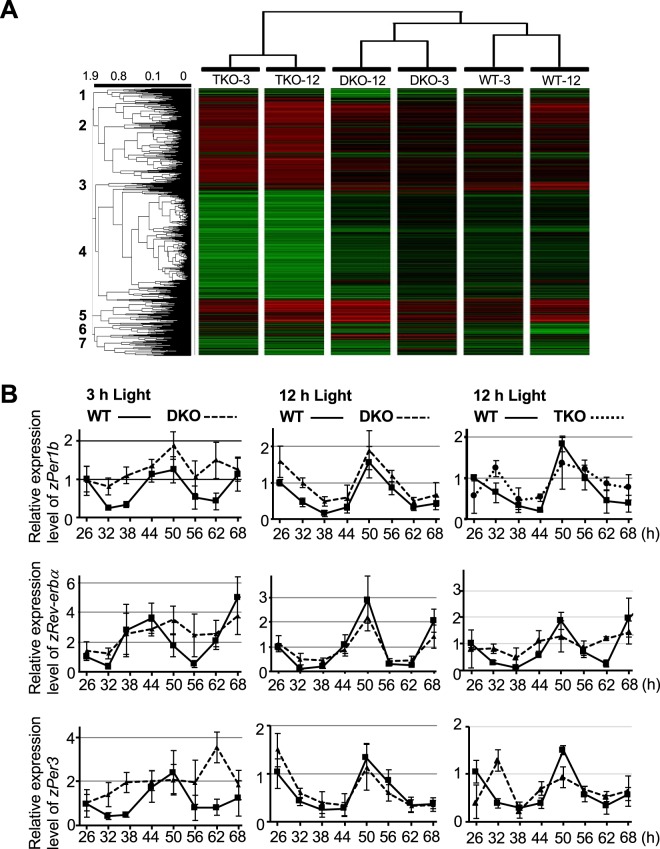
Analysis of clock gene expression profiles in WT, DKO, and TKO zebrafish. (**A**) Heatmap analysis of microarray data showing hierarchical clustering of genes. The expression levels in zebrafish exposed to 3-h and 12-h light were first normalized against those in animals kept in the darkness for WT, DKO, or TKO zebrafish. The normalized expression levels were then compared among animals of the three different genotypes. Red or green colors indicate differentially up- or downregulated genes, respectively. The dendrogram displayed on top was based on hierarchical clustering of the samples using the average linkage method. (**B**) Left and middle panels: WT (solid line) and DKO (dotted line) zebrafish were raised in DD conditions and exposed to 3-h (left panel) or 12-h (middle panel) light at the beginning of 6 dpf. Expression levels of indicated genes were examined by RT-PCR analysis at the indicated time points after the treatment. Right panels: WT (solid line) and TKO (dotted line) zebrafish were raised in DD conditions and exposed to 12-h light at the beginning of 6 dpf. Expression levels of indicated genes were examined by RT-PCR analysis at indicated time points after the treatment. In all experiments, the value at time point 0 was set as 1 for each gene. Values are mean ± S.E.M. of three independent experiments.

Next, we performed a microarray analysis to identify the underlying circadian mechanism causing the defects in light-dependent behavioral rhythm formation in DKO and TKO zebrafish (Fig. 4A). We prepared nine samples from WT, DKO, or TKO animals treated with 3-h light, 12-h light, and those kept in darkness. To clarify the light-dependent gene expression profiles in animals of each genotype, the gene expression levels in WT, DKO, and TKO zebrafish treated with 3- or 12-h light were normalized against the corresponding genotype kept in darkness. The normalized expression levels were then compared between animals of the three different genotypes. Cluster analysis of the microarray results identified seven clusters (Supplementary Fig. S7A). We identified target genes of interest whose expression levels change in the same direction between zebrafish that form behavioral rhythms (i.e. WT and DKO animals exposed to 12-h light) and those that do not (i.e. DKO animals exposed to 3-h light, and TKO animals exposed to 3-h or 12-h light). Accordingly, we focused on genes in cluster 6, which were efficiently inhibited in animals that establish behavioral rhythm relative to those with defective behavioral rhythms, and included a number of cellular clock-target genes (Supplementary Table S2). As expected, pathway analysis suggested that cluster 6 genes regulate “CLOCK:BMAL-dependent transcription” (Supplementary Fig. S7B). To confirm the result of the pathway analysis, we used RT-PCR to examine expression profiles of the CLOCK:BMAL target genes *zPer1b*, *zRev-erbα*, and *zPer3* in zebrafish (Fig. 4B). We found that a 3-h light exposure induced circadian expression of these genes in WT zebrafish, but not in DKO animals, whereas a 12-h light exposure induced circadian gene expression in both DKO and WT zebrafish. In contrast, 12-h light failed to establish circadian oscillation of these genes in TKO zebrafish. These results were confirmed by the cosinor analysis (Supplementary Table S3). Our statistical analysis with Two Way ANOVA’s method revealed increased expression of *zPer1* and *zPer3* in DKO animals exposed to 3-h and/or 12-h light (Supplementary Table S4). These results reveal that circadian gene expression was not established in zebrafish with defective behavior rhythm formation (Supplementary Table S5). Thus, our results suggest that the defect in formation of behavioral rhythms in DKO and TKO zebrafish is due to deregulation of their cellular clocks.

### Light-dependent synchronization of cellular clocks is impaired in TKO cells

**Figure 5:**
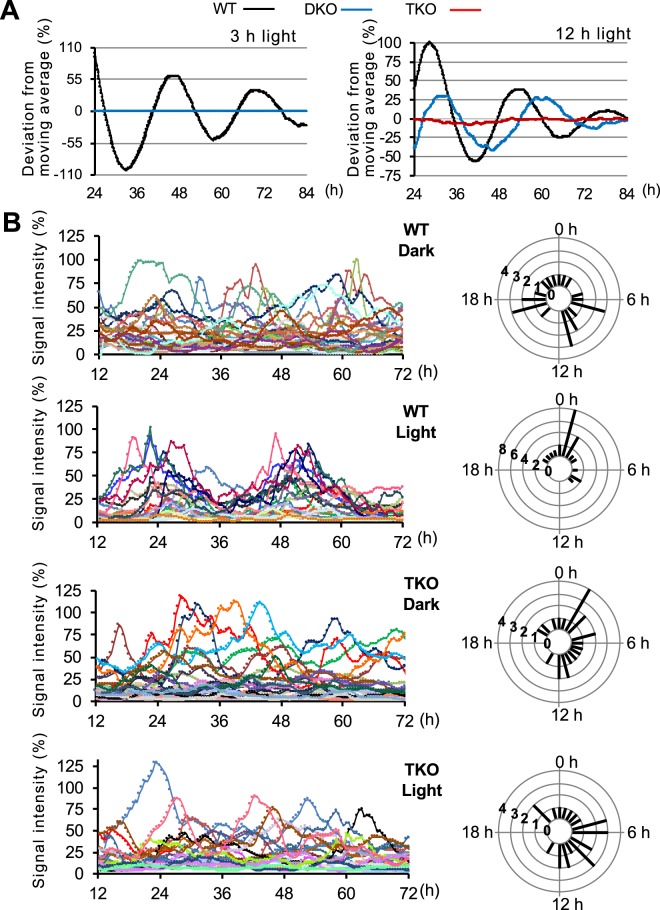
Characterization of cellular clocks in WT and TKO cells at the single cell level. (**A**) WT, DKO, and TKO cultured cells were transfected with the *Per1*::Luc construct and exposed to light for 3-h or 12-h. *Per1* reporter bioluminescence in each cultured cell was monitored over the indicated time course. The bioluminescence values were detrended according to the instrument protocol and the detrended values were normalized by averaging intensity over time. Detrended data representative of three independent experiments are shown. (B) Left panels: Luminescent traces from individual WT and TKO cultured cells maintained in DD conditions (Dark) or exposed to light for 12-h (Light) were monitored by real-time bioluminescence imaging. [WT dark (n = 24), WT light (n = 24), TKO dark (n = 23) TKO light (n = 25)] Right panels: Circular histograms of phase distribution from WT or TKO cultured cells kept in DD conditions (Dark) or exposed to 12 h light (Light). The times having peak signal levels were determined using the “Cosinor” and “Acro” software.

We next investigated the mechanism underlying the impaired light-dependent regulation of cellular clocks. To this end, we used zebrafish cultured cells, a well-known cell-based experimental system for studying the effects of light on cellular clocks^14,15^. We established cell cultures from WT, DKO, and TKO zebrafish embryos, and analyzed circadian transcription by real-time monitoring of a *Per1* clock gene promoter-driven firefly luciferase reporter (Fig. 5A and Supplementary Fig. S8). A 3-h light exposure triggered a circadian oscillation of firefly luciferase activity in WT cultured cells but not in DKO cells, whereas exposure with 12-h light established the circadian change of bioluminescence in DKO and WT cells but not in TKO cells. Thus, the light exposure and genotypes required to establish the circadian oscillation of gene expression were highly similar in zebrafish cells *in vitro* (Fig. 5A) and in zebrafish *in vivo* (Fig. 4B).

The impaired circadian oscillation of gene expression in DKO and TKO cells could be explained either by arrhythmicity of each cellular clock, or by the impaired synchronization of rhythmic cellular clocks. To discriminate between these two possibilities, we developed a zebrafish cell-based system in which rhythmic expression of the *Per1* promoter-driven bioluminescence could be detected at the single cell level. We chose nanoluciferase (Nluc) as a reporter because the bioluminescence intensity of Nluc is about 100-fold higher than that of firefly luciferase^37^. To allow for a large amplitude of cyclic Nluc accumulation, we engineered a short-lived bioluminescent Nluc fusion protein with a C-terminal PEST element. We used the *Per1*::Nluc-PEST construct to generate *Per1*::Nluc reporter WT and TKO cells (Nluc-WT and Nluc-TKO cells).

First, we conducted real-time recording of bioluminescence from single Nluc-WT cells, which were kept in the dark for 2.5 days prior to recording (Fig. 5B). The bioluminescence profile revealed that, as expected, individual cells displayed a robust but out-of-phase circadian oscillation of Nluc signal intensity. Circular histograms of phase distribution confirmed this random distribution of phase. Next, we kept Nluc-WT cells kept in constant dark conditions for 2 days, exposed them to 12-h light, and recorded their bioluminescence in constant dark conditions. We found that the entire population of individual luminescent cells oscillated in a common phase of the circadian cycle after the 12-h light exposure. The circular histograms of phase distribution demonstrated that Nluc-WT cells exposed to light maintained a common phase.

Next, we conducted single cell bioluminescence recording using Nluc-TKO cells kept in constant dark conditions for 2.5 days. Bioluminescence intensities in Nluc-TKO cells displayed a clear but out-of-phase circadian oscillation in constant dark conditions, suggesting that loss of *zCry1a*, *zPer2*, and *zCry2a* does not lead to arrhythmicity of each cellular clock. Importantly, exposure to 12-h light did not synchronize the phases of circadian oscillation of bioluminescence in Nluc-TKO cells. Circular histograms of phase distribution confirmed the defective light synchronization of bioluminescence oscillation. Taken together, our results suggest that the impaired formation of behavioral rhythms in DKO and TKO zebrafish larvae is not due to the inactivation of individual cellular clocks, but instead reflects a defective light-dependent synchronization of cellular clocks.

### Reduced activity of DKO and TKO zebrafish is linked to abnormal regulation of metabolism

**Figure 6:**
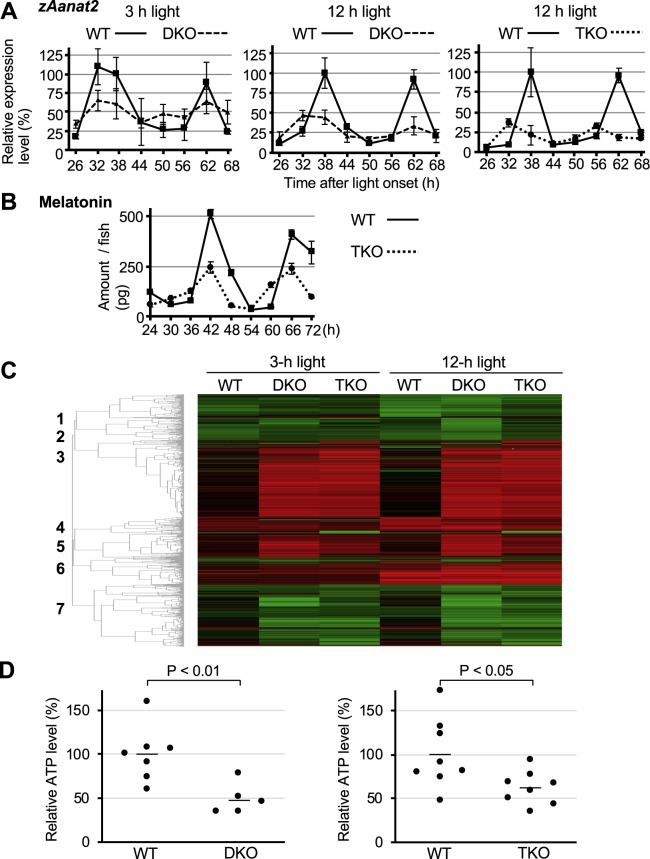
Identification of molecular mechanisms underlying the reduced activity of DKO and TKO zebrafish. (**A**) Left and middle panels: WT (solid line) and DKO (dotted line) zebrafish were raised in DD conditions and exposed to 3-h (left panel) or 12-h (middle panel) light at the beginning of 6 dpf. The expression level of zAanat2 was examined by RT-PCR analysis. Right panel: WT (solid line) and TKO (dotted line) zebrafish were raised in DD conditions and exposed to 12-h light at the beginning of 6 dpf. The expression level of *zAanat2* was examined by RT-PCR analysis. In all experiments, the value at time point 0 was set to 1. Values are mean ± S.E.M. of three independent experiments. (**B**) Zebrafish were raised in DD conditions and exposed to a 12-h light pulse at the beginning of 6 dpf. At indicated time points after the onset of light, melatonin levels in each WT (solid line) and TKO (dotted line) zebrafish were examined by LC-MS/MS. Values are mean ± S.E.M. of three independent experiments. (**C**) Heatmap analysis of microarray data showing hierarchical clustering of differentially expressed genes in WT zebrafish kept in darkness relative to WT, DKO, and TKO zebrafish exposed to 3-h or 12-h light. Red or green colors indicate differentially up- or downregulated genes, respectively. Hierarchical cluster analysis of the microarray data identified seven gene clusters. (**D**) Measurement of ATP levels in WT, DKO and TKO zebrafish. WT, DKO and TKO animals raised in constant darkness were exposed to light for 12-h at the beginning of 6 dpf. They were then kept in constant darkness for 12-h and prepared for the ATP assay. In both experiments for DKO and TKO zebrafish, the average ATP level in WT zebrafish was set to 100%. Left panel; WT (n = 7), DKO (n = 5). Right panel; WT (n = 8), TKO (n = 8). P < 0.01 and P < 0.05 (Student’s t-test).

Next, we focused on identifying the molecular mechanism underlying the reduced locomotor activity in the DKO and TKO zebrafish. The sleep-inducing hormone melatonin has been reported to suppress activity in zebrafish^13,38^. We predicted that melatonin levels would increase in DKO and TKO zebrafish. To test this hypothesis, we exposed DKO and TKO zebrafish to 3- or 12-h of light and examined the levels of mRNA encoding the melatonin synthase *zAanat2*^12,39^. Contrary to our expectations, *zAanat2* mRNA levels were reduced in DKO and TKO animals compared to those in WT animals (Fig. 6A,B). Further, TKO animals exposed to 12-h light displayed reduced melatonin protein levels compared to similarly treated WT animals. Thus, the reduced activity of DKO and TKO zebrafish does not arise from up-regulation of melatonin.

We then conducted a microarray analysis to gain insight into the molecular mechanism underlying the reduced activity of DKO and TKO zebrafish compared to WT zebrafish (Fig. 6C). We prepared samples from WT, DKO, and TKO animals exposed to 3-h and 12-h light, and normalized the gene expression levels to WT animals kept in darkness. Cluster analysis identified seven gene clusters (Supplementary Fig. S9). Our earlier results showed that the reduced activity depended on disruption of both *zCry1a* and *zPer2* genes, and not on the lighting conditions (Fig. 1B,C, Supplementary Table S5). Thus, we reasoned that the expression levels of the genes maintaining activity likely change in a common direction in DKO and TKO zebrafish when compared to WT animals, regardless of the light conditions. Accordingly, we focused on clusters 3 and 7: genes in cluster 3 showed elevated expression in DKO and TKO zebrafish compared to in WT animals, whereas genes in cluster 7 showed reduced expression in DKO and TKO zebrafish compared to in WT animals (Supplementary Fig. S10). We performed a gene ontology (GO) analysis and uncovered GO terms related to metabolism, such as “metabolic process” and “single-organism metabolic process”, in clusters 3 and 7, but not in the other clusters (Supplementary Fig. S11). The genes constituting the GO terms related to metabolism in clusters 3 and 7 included those regulating cellular ATP levels (Supplementary Fig. S10). ATP is an important cellular energy carrier, and thus its level tightly relates to the physical activity of the animal. We used a firefly luminescence-based ATP assay system to measure ATP levels in WT, DKO, and TKO zebrafish exposed to 12-h light (Fig. 6D). Notably, ATP levels in DKO and TKO zebrafish were significantly lower than that in WT animals. We found that glycolytic genes such as *Hexokinase domain containing 1*, *6-phosphofructo-2-kinase/fructose-2, 6-biphosphatase 2a* (*Pfkfb*) and *Phosphoglucomutase 1* in cluster 3 were down-regulated in DKO and TKO animals. Taken together, our results suggest that impaired cellular energy metabolism contributes to the reduced activity of DKO and TKO zebrafish.

## Discussion

Our work shows that zebrafish use multiple light-induced cellular clock regulators for the light-dependent synchronization of cellular clocks. In nature, zebrafish are exposed to light-dark cycles and the intensity of light depends on the time of day and local conditions. The existence of multiple cellular clock regulators might allow the zebrafish circadian clock to benefit from different qualities of external light. Further, cross-talk between acute (zPER2 and zCRY1a) and slow (zCRY2a) light-induced cellular clock regulators might tightly synchronize cellular clocks to confer robustness to the zebrafish circadian clock in the face of a wide range of environmental conditions.

Mouse *Per1* and *Per2* genes are induced by the CLOCK (NPAS2):BMAL complex via E-box and by light via cAMP responsive element^40^. The CLOCK(NPAS2):BMAL-dependent induction of *Per1* and *Per2* is essential for formation of the cellular clock, whereas the light-dependent induction of *Per1* and *Per2* is proposed to be involved in the light-dependent synchronization of cellular clocks. However, whether light-dependent induction of *Per1* and *Per2* is required to synchronize cellular clocks has not been fully elucidated by adequate genetically modified mice. Genetic inhibition of both mouse *Per1* and *Per2* genes disrupts the cellular clock, and thus prevents the analysis of synchronization^41^.

In zebrafish, there are four transcriptional repressor type *Cry* genes (*zCry1a*, *zCry1b*, *zCry2a*, and *zCry2b*) and four *Per* genes (*zPer1a*, *zPer1b*, *zPer2*, and *zPer3*). *zCry2a* and *zCry2b* are induced by both the CLOCK (NPAS2):BMAL complex and by light; *zCry1b*, *zPer1a*, *zPer1b*, and *zPer3* are induced by the CLOCK (NPAS2):BMAL complex but not by light; and *zCry1a* and *Per2* are induced by light but not by the CLOCK (NPAS2):BMAL complex^21,26,28,31,33^. These distinct dependencies of *zPer* and *zCry* gene expression allowed us to uncover a role for light-induced zPER2, zCRY1a and zCRY2a in the light-dependent synchronization of cellular clocks. Thus, zebrafish is a particularly useful animal model for studying the light-dependent synchronization of cellular clocks.

In mammals, light signal is received by the retina and then integrated to the SCN cellular clocks^8,9^. The SCN cellular clocks then transmit light information to peripheral cellular clocks via humoral signals, and synchronize them. Furthermore, recent studies have reported that factor(s) other than cellular clocks in SCN can synchronize peripheral cellular clocks in a light-dependent manner^42^. In contrast, in zebrafish, light directly synchronizes peripheral cellular clocks in addition to central cellular clocks^14,15^. Hence, there are two possible mechanisms for synchronization of zebrafish peripheral cellular clocks: a central clock-dependent mechanism and a light-regulated mechanism. The whole-body KO animals generated in the current study cannot distinguish whether the synchronization of peripheral clocks is disrupted due to defects in central clocks or in light-dependent regulation. Future analyses of central or peripheral-specific KO zebrafish of *zPer2*, *zCry1a* and *zCry2a* would be informative on this point.

We found that zPER2 and zCRY1a regulate the expression of genes controlling energy metabolism, which likely contributes to the locomotor activity of zebrafish. zPER2 and zCRY1a form a heterodimer that functions as a transcriptional regulator^43^. As mentioned above, the activity control by zPER2 and zCRY1a is independent of the circadian clock. Therefore, the zPER2:zCRY1a complex would not target the CLOCK (NPAS2):BMAL complex to control energy metabolism (Supplementary Fig. S12). Previous studies in mice and zebrafish have reported that PER2 physically interacts with nuclear receptors, such as PPARα, REV-ERBα, RORα and PPARγ, to regulate their transcriptional activities^27,44,45^. In particular, PER2-mediated regulation of PPARγ‘s transcriptional activity contributes to the control of mouse lipid metabolism^45^. Based on these facts, we predict that the zPER2:zCRY1a complex directly and/or indirectly regulates the activities of various transcription factors whose targets play key roles in controlling cellular metabolism, thereby maintaining activity. Components of the circadian clock regulate both behavioral rhythms and locomotor activity, which likely functions to maximize physiological efficiency.

Finally, our data show that zPER2 and zCRY1a regulate cellular ATP level. We speculate that impaired glycolysis could underlie the decreased ATP levels in DKO and TKO zebrafish. It has been reported that hypoxia-inducible factor 1α (HIF-1α) regulates glycolysis genes, including *Pfkfb* and *Phosphoglucomutase 1*, under both normoxic and hyperoxic conditions^46–48^. Recently, HIF-1α was shown to interact with circadian clock regulators in zebrafish and mammals^49–51^. BMAL1 interacts with HIF-1α to regulate the expression of HIF-1α target genes^52,53^. Thus, it is tempting to speculate that zCRY1a and zPER2 regulate the activity of the BMAL1-HIF-1α complex to control the expression of glycolysis genes and cellular ATP levels.

## Materials and Methods

### Statement on the Ethical Treatment of Animals

This study was carried out in strict accordance with the recommendations in the ethical guidelines of Tokyo Medical and Dental University (TMDU). All experimental protocols in this study were approved by the Animal Welfare Committee of TMDU (Permit Number: A2017-048C2). All experiments were performed in a manner that minimized pain and discomfort.

### Fish

The TL zebrafish were maintained in distilled water, to which a small amount of salts and minerals is added. The water was heated to 26 °C, filtered and recycled continuously. Zebrafish were fed twice daily. Embryos and young zebrafish were raised and maintained in Egg water at 28 °C in an incubator, as described previously^54^.

### Generation of KO zebrafish

TALEN was used to generate *zCry1a* KO and *zPer2* KO zebrafish. The plasmids for synthesizing TALEN mRNAs were constructed in a two-step assembly system as described previously^55,56^. Six or fewer TAL effector repeat modules were ligated into pFUS vectors (intermediate array vectors) as the first step. Subsequently, the intermediate array vectors and last TAL effector repeat were ligated into the pCS2TAL3DD vector for a forward TALEN or the pCS2TAL3RR vector for a reverse TALEN as the second step. The TALEN mRNAs were transcribed from the linearized above-mentioned plasmids using T7 polymerase (Toyobo). To generate *zPer2* and *zCry1a* KO zebrafish, forward and reverse TALEN mRNAs (400 pg each) targeting the *zPer2* and *zCry1a* genes, respectively, were injected into the blastomeres of one-cell stage WT zebrafish embryos as described previously^57^. The produced *zPer2* KO zebrafish and *zCry1a* KO zebrafish were then crossed to generate DKO zebrafish.

The CRISPR/Cas9 system was used to disrupt the *zCry2a* and *zCry2b* genes. To construct the single-guide RNA (sgRNA)-expression plasmids, pDR274-*zCry2a* and pDR274-*zCry2b*, sense and anti-sense oligonucleotides targeting *zCry2a* or *zCry2b* were annealed and inserted into the BsaI-cleaved pDR274 vector. The *Cas9* expression plasmid pCS2-hSpCas9 was purchased from Addgene. The sgRNAs were transcribed from the linearized pDR274-*zCry2a* and pDR274-*zCry2b* using T7 polymerase (Toyobo). The *Cas9* mRNA was transcribed from the linearized pCS2-hSpCas9 using mMESSAGE SP6 Kits (Thermo Fisher Scientific). To generate TKO zebrafish, Cas9 mRNA (200 pg) and sgRNA (50 pg) targeting *zCry2a* were injected into the blastomeres of one-cell stage DKO zebrafish embryos.

To check the genotypes of the produced KO zebrafish, zebrafish fin clips were incubated in 50 μl of lysis buffer (10 mM Tris-HCl (pH 8.0), 1 mM EDTA, 0.2% Triton X-100, and 200 μg/ml proteinase K) at 50 °C for 8 h. Then, the solution was incubated at 100 °C for 10 min to inactivate proteinase K. The TALEN and CRISPR target loci were amplified using the supernatants as templates and the following primers: zCry1a forward, 5′-GCTCTAGATCAATACAGTCCACTGGTTCAGGA-3′ and zCry1a reverse, 5′-GCGGTACCCGAGGATGTACACACAGCGGACAC-3′ for the *zCry1a*-TALEN target locus; zPer2 forward, 5′-GCGGTACCCCACAACACCCGGAAGATGA-3′ and zPer2 reverse, 5′-GCTCTAGATCATTGCCATGAGACTCTGTTCC-3′ for the *zPer2*-TALEN target locus; zCry2a forward, 5′-TCCAGGTTGACCTTCGAGTATGA-3′ and zCry2a reverse, 5′-AAAGGGTGTGTGAGGTTTTGACA-3′ for the *zCry2a*-sgRNA target locus; zCry2b forward, 5′-CAGCAGTAAATAATAGGAGCGTTTG-3′ and zCry2b reverse, 5′-GCTCCATTCGCTTCAGAGCTTC-3′ for the *zCry2b*-sgRNA target locus. PCR products were electrophoresed on 15% polyacrylamide gels (Nacalai tesque). All full gel electrophoresis images are shown in Supplementary Fig. S13.

### Analysis of zebrafish behavior

Fertilized eggs were kept in a temperature-controlled incubator at 28 °C and maintained under constant darkness before light treatment. 5 dpf zebrafish were then placed in 48-well plates, exposed to light and transferred to constant dim light conditions. Locomotor activities of zebrafish were tracked by the DanioVision Tracking System (Noldus Information Technology) and analyzed by the Ethovision 8.0 software (Noldus Information Technology). Tracks were analyzed for the total distance moved by each larva per 10 min time-bins. Evaluation of circadian rhythmicity in the zebrafish’s behavior was conducted by ActogramJ software^58^.

For the resting behavior analysis, movement distance data of each 1 sec time-bin were generated by Ethovision software and the bins were classified into active (with movement) and immobile (without a detectable movement). Continuous active bins and continuous immobile bins were defined as active bouts and rest bouts, respectively. Then, using a custom-made program by R^59^, the lengths of active bouts and rest bouts were calculated and the cumulative probability distribution plots of summed up data of each genotype were generated (Supplementary Fig. S6). Next, the parameters in Table 2 were calculated according to the definition of resting as a 60 sec or longer rest bout.

### Quantitative real-time (RT)-PCR

Total RNA extraction was carried out using TRIzol (Invitrogen) according to the manufacturer’s instructions. Total RNA was reverse-transcribed into cDNA using Superscript III Reverse Transcriptase (Invitrogen) and oligo (dT) primer. Each quantitative real-time RT-PCR reaction was performed using the Chromo4 real-time detection system (Bio-Rad). For a 20 μl PCR reaction, 10 μl containing cDNA template mixed with the appropriate primers to a final concentration of 200 nM was combined with 10 μl iQ SYBR Green Supermix (Bio-Rad). The reaction was incubated at 95 °C for 3 min, followed by 50 cycles at 95 °C for 30 s, 60 °C for 30 s, and 72 °C for 30 s. *zActin* gene was used for normalization. In order to assess circadian rhythmicity of each gene expression, cosinor analysis was carried out using Cosinor software (Circadian Rhythm Laboratory of Boise State University, Boise, ID, USA). PCR primer sequences used in the current study are listed in Supplementary Table S6.

### Melatonin measurement

Zebrafish were added to 100 µl of Milli-Q water and homogenized on ice. Subsequently, 4 volumes of acetone were added and vortexed for 5 min, followed by centrifugation at 18000 G for 10 min. Supernatants were then transferred into clean test tubes and evaporated to dryness at 65 °C under a stream of nitrogen gas. After dissolving residues into 100 µL of Milli-Q water, mixtures were filtered through filters with 0.22 μm pores (Centrifugal Filter Units Ultrafree-MC-GV 0.22 μm, Merck Millipore, Guyancourt, France) and stored at −80 °C until liquid chromatography-tandem mass spectrometry (LC-MS/MS) analyses.

Melatonin was analyzed using LC-MS/MS. Briefly, 10 µL samples were injected into a HPLC system (AC30AD, Shimadzu Corporation, Kyoto, Japan) equipped with a C18 2.0 × 150-mm, 3 µm Kinetex column (Tosoh, Japan). The mobile phase comprised 10 µM ammonium acetate in 0.05% (v/v) acetic acid with varying concentrations of MeOH. The linear gradient was run over 20 min from 5% to 50% MeOH and was then maintained at 100% MeOH for 10 min. The flow rate was 0.3 mL/min and the auto sampler and column oven were maintained at 4 °C and 25 °C, respectively. Melatonin was detected using a triple quadrupole mass spectrometer (LCMS-8050, Shimadzu) and quantified using a multiple reactions monitoring MRM method with transitions of parent ions to product ions. The transition for melatonin was m/z 233.0–130.0. Limits of sensitivity for melatonin was 11.1 fg for a 2:1 signal-to-noise ratio. Intra- and inter-assay variation coefficients were 3.94% and 4.60%.

### Microarray analysis

The microarray analysis was entrusted to Takara Bio Inc. (Japan). Total RNA was extracted from zebrafish treated as described in figure legends using Trizol Reagent (Invitrogen). The quality of RNA was initially assessed by electrophoresis on a 1.5% agarose gel, and further by absorption spectrophotometry (Agilent Bioanalyser 2100; Agilent, Palo Alto, CA, USA). cDNAs were synthesized by the Low Input Quick Amp Labelling Kit. Cy3-labelled cRNA was synthesized by *in vitro* transcription with T7 RNA polymerase. Following fragmentation, cRNA was hybridized on the zebrafish microarray. GeneChips were washed and scanned. Microarray data were processed using GeneChip Operating Software. The gene ontology or pathway analyses were conducted by the KeyMolnet Software (Version 2015; Institute of Medicinal Molecular Design Inc.). Gene expression microarray data have been deposited in the GEO database, with accession number GSE111521. These can be reviewed at: https://www.ncbi.nlm.nih.gov/geo/query/acc.cgi?acc=GSE111521 by entering token ihermqaqplkbbsd in the box.

### Real-time bioluminescence assay

Zebrafish cultured cells were prepared from WT, DKO, or TKO embryos as described previously^60^. They were cultured at 28 °C in L-15 medium (Sigma) containing 15% fetal bovine serum. Zebrafish cultured cells were transfected with plasmids and synchronized with light as indicated in the figure legends. Real-time bioluminescence in the cells with 0.2 mM luciferin (Toyobo, Japan) was monitored using Kronos (ATTO, Japan), as described previously^61^. If the Y-axis indicates “deviation from the moving average,” the values were detrended according to the instrument protocol (Kronos; ATTO, Japan). All detrended values were normalized by averaging intensity over time. The data in the graph were further normalized using maximum circadian peak intensities over time.

### Generation of zebrafish cells expressing nanoluciferase reporter under the control of the *Per1* promoter

The retrovirus infection system (IMGENEX, San Diego, CA) was used to transduce the Nluc-based clock reporter into zebrafish cells. To construct the retrovirus vector expressing Nluc fused to the PEST sequence (Nluc-PEST) under the control of the mouse *Per1* promoter, the CMV promoter sequence of pCLNCX vector was first replaced with the *Per1* promoter sequence (pPer1-CLNCX). The DNA sequence of Nluc-PEST was then ligated into the pPer1-CLNCX (pPer1-Nluc-PEST-CLNCX). The Per1 promoter-driven Nluc-PEST was expressed in WT or TKO zebrafish cultured cells using the pPer1-Nluc-PEST-CLNCX, with pMD.G/vsv-g as the enveloping vector to produce the Nluc-WT and Nluc-TKO cells.

### Single cell imaging analysis

Real-time bioluminescence in the Nluc-WT and Nluc-TKO cells was monitored at the single cell level using an LV200 (Olympus, Japan) as described previously^62^. EnduRen (Promega) was used as the substrate of NLuc. Values were normalized to maximum peak intensity of sample of WT cells kept in the darkness over time and to average intensity over time. To test the significance of the circadian rhythmicity and acrophase, we performed computerized analysis of normalized data in “Cosinor” and “Acro” software downloaded from the Circadian Rhythm Laboratory Software home page (http://www.circadian.org/softwar.html).

### Phylogenetic tree

Amino acid sequences of PER and CRY of various species were obtained from the Ensembl database. The phylogenetic tree was constructed using the neighbor-joining method and ClustalX software^63^. The reliability of the tree was estimated using the bootstrap method and 10,000 replications.

### Measurement of ATP levels

ATP levels in zebrafish were measured using a luminescence-based ATP assay kit (Toyobo) according to the manufacturer’s instructions. In brief, 6 dpf zebrafish exposed to light as indicated in the legend of Fig. 6D were soaked in the ATP extraction reagent. The supernatants were mixed with the L/L reagent, which contains luciferase and D-luciferin, and produces luminescence in the presence of ATP. Luciferase activities from the mixtures were quantified by means of a luminometer.

## Supplementary information

Supplementary Information
