# Supplementary material for: The clock components Period2, Cryptochrome1a, and Cryptochrome2a function in establishing light-dependent behavioral rhythms and/or total activity levels in zebrafish
Source: Sci Rep. 2019 Jan 17;9:196. doi: 10.1038/s41598-018-37879-8 (PMC6336812; doi:10.1038/s41598-018-37879-8)
Supplement: Supplementary file 1 — Supplementary Information [file 41598_2018_37879_MOESM1_ESM.pdf]

Supplementary information

The clock components *Period2*, *Cryptochrome1a*, and *Cryptochrome2a* function in establishing light-dependent behavioral rhythms and/or total activity levels in zebrafish

Jun Hirayama[1, 2], Yikelamu Alifu[1], Rin Hamabe[1], Sho Yamaguchi[3], Jun Tomita[3], Yusuke Maruyama[4], Yoichi Asaoka[5], Ken-ichi Nakahama[6], Teruya Tamaru[7], Ken Takamatsu[7], Nobuhiko Takamatsu[8], Atsuhiko Hattori[4], Sachiko Nishina[9], Noriyuki Azuma[9], Atsuo Kawahara[10], Kazuhiko Kume[3], Hiroshi Nishina[1]

1. Department of Developmental and Regenerative Biology, Medical Research Institute, Tokyo Medical and Dental University (TMDU), Tokyo, Japan
2. Department of Clinical Engineering, Faculty of Health Sciences, Komatsu University, Ishikawa, Japan
3. Department of Neuropharmacology, Graduate School of Pharmaceutical Sciences, Nagoya City University, Nagoya, Japan
4. Department of Biology, College of Liberal Arts and Sciences, Tokyo Medical and Dental University (TMDU), Ichikawa, Japan
5. Department of Microbiology and Immunology, Yamaguchi University Graduate School of Medicine, Ube, Japan.
6. Department of Cellular Physiological Chemistry, Tokyo Medical and Dental University (TMDU), Tokyo, Japan.
7. Department of Physiology and Advanced Research Center for Medical Science, Toho University School of Medicine, Tokyo, Japan
8. Department of Biosciences, School of Science, Kitasato University, Sagamihara, Japan
9. Department of Ophthalmology and Laboratory for Visual Science, National Center for Child Health and Development, Tokyo, Japan.
10. Laboratory for Developmental Biology, Center for Medical Education and Sciences, Graduate School of Medical Science, University of Yamanashi, Yamanashi, Japan.

Supplementary figure legends

Supplementary Fig. S1. Genome editing patterns of *zCry1a* and *zPer2* genes.

(A) Polymerase chain reaction (PCR) products of *zCry1a* or *zPer2* genes from the genomic DNAs of WT, *zPer2* KO, *zCry1a* KO and DKO zebrafish were electrophoresed on a 15% polyacrylamide gel. (B) Upper panel: TALEN-induced mutation pattern of the *zCry1a* gene. The deleted nucleotides in the DNA sequences are indicated by dashes. The WT sequence is shown at the top. TALEN recognizing and spacer sequences are indicated by capital and small letters, respectively. Lower panel: TALEN-induced mutation pattern of the *zPer2* gene. The deleted nucleotides in the DNA sequences are indicated by dashes. The WT sequence is shown at the top. TALEN recognizing and spacer sequences are indicated by capital and small letters, respectively.

Supplementary Fig. S2. Behavioral patterns in zebrafish kept in DD condition.

Representative double-plotted activity records (actograms) of WT zebrafish kept in DD condition. WT zebrafish were raised in DD conditions for 5 dpf, and their behavior was then analyzed in DD condition. The ratio (%) of rhythmic animals was 12.5 % (n = 8).

Supplementary Fig. S3. Schematic of Experimental Setup for Fig 2C, Supplemental Fig S4.

Animals were raised in DD condition after fertilization until the beginning of 6 dpf, then exposed to 0-h, 3-h or 12-h of light. Larvae were harvested for RNA every 3 h (starting from the onset of light, t=0), as indicated by a *. Black and white bars represent dark and light conditions, respectively.

Supplementary Fig. S4. Gene expression patterns in zebrafish kept in DD condition.

A time course of clock gene expression. Zebrafish larvae were raised in the darkness and harvested for RNA every 3 h starting from the beginning of 6 dpf until 6.5 dpf. RT-PCR was performed for the indicated genes. The value at time point 0 was set as 1

for each gene. Values are mean ± S.E.M. of three independent experiments. Experimental conditions were the same as those in Fig. 2C except without light treatment (see Supplemental Fig. S3). *zCry2a*, *zCry2b*, *zPer2* and *zCry1a* were induced in animals treated with light (Fig. 2C), but not in animals kept in DD condition (Supplementary Fig. S4), showing that induction of these genes is light-dependent.

Supplementary Fig. S5. Genome editing patterns of *zCry2a* and *zCry2b* genes.

(A) High mobility assay (HMA) using genomic DNAs isolated from embryos injected with *Cas9* mRNA and sgRNAs targeting *zCry2a* or *zCry2b* in a 15% polyacrylamide gel. Left: schematic diagram of electrophoresis patterns for the targets' PCR products with (+) or without (-) genome edits. Sample with genome edit shows multiple PCR products, which result from heteroduplexes. Middle: *Cas9* mRNA and sgRNAs targeting *zCry2a* or *Gfp* were injected into WT or DKO zebrafish embryos, and genomic DNAs were isolated from the injected (+ *zCry2a* or *Gfp* sgRNA) or non-injected embryos (No sgRNA). Genome edits in the target locus were evaluated by HMA profiles of the PCR products. Right: *Cas9* mRNA and sgRNAs targeting *zCry2b* or *Gfp* were injected into WT or DKO zebrafish embryos, and genomic DNAs were isolated from the injected (+ *zCry2b* or *Gfp* sgRNA) or non-injected embryos (No sgRNA). Genome edits in the target locus were evaluated by HMA profiles of the PCR products. (B) *Cas9* mRNA and sgRNAs targeting *zCry2a* were injected into WT zebrafish embryos, and genomic DNAs were isolated from the injected (+) or non-injected embryos (-). Genome edits in the target locus were evaluated by HMA profiles of the PCR products. The time of electrophoresis was sufficient to distinguish the mutated PCR products from the WT PCR products. (C) CRISPR-induced mutation pattern of the *zCry2a* gene. The deleted nucleotides in the DNA sequences are indicated by dashes. The WT sequence is shown at the top. (D) The PCR products of *zPer2*, *zCry1a*, and *zCry2a* genes from the genomic DNAs of WT and TKO zebrafish were electrophoresed on a 15% polyacrylamide gel.

Supplementary Fig. S6. Distribution analysis of rest bout length.

Double logarithmic plots of the cumulative probability of the rest bout for the combined data of WT, DKO and TKO zebrafish [WT (n = 12), DKO KO (n = 12), TKO

(n = 10)]. Dashed line indicates 60 sec.

Supplementary Fig. S7. Identification of candidate biological functions involved in light-dependent formation of behavioral rhythms in zebrafish. (A) The average expression levels of genes in three different genotypes (W: WT, D: DKO and T: TKO) of animals exposed to 3-h or 12-h light are shown for each of the seven gene clusters identified in Figure 4A. The number of genes averaged in each cluster are 231 (cluster 1), 210 (cluster 2), 738 (cluster 3), 163 (cluster 4), 232 (cluster 5), 256 (cluster 6), and 592 (cluster 7). (B) Candidate pathways involved in light-dependent formation of zebrafish's behavioral rhythms were identified by pathway analysis with genes included in cluster 6. The pathway analysis was conducted by the KeyMolnet Software (Version 2015; Institute of Medicinal Molecular Design Inc.).

Supplementary Fig. S8. Characterization of cellular clocks in WT, DKO and TKO cultured cells. WT, DKO, and TKO cultured cells were transfected with the *Per1*-promoter firefly luciferase reporter plasmid, and exposed to light for 3-h (upper panel) or 12-h (lower panel). *Per1* reporter bioluminescence was monitored over the indicated time course. The raw bioluminescence data (mean ± S.E.M. of three independent experiments) are shown.

Supplementary Fig. S9. Characterization of seven gene clusters described in Figure 6C. The number of genes averaged in each cluster is 203 (cluster 1), 1143 (cluster 2), 82 (cluster 3), 1608 (cluster 4), 324 (cluster 5), 141 (cluster 6), and 310 (cluster 7).

Supplementary Fig. S10. Summary of the microarray data-analyzing processes to identify the candidate mechanism underlying the reduced activity of DKO or TKO zebrafish.

Supplementary Fig. S11. Identified biological functions by the gene ontology analyses using genes in each of the seven clusters shown in Figure 6C.

Supplementary Fig. S12. A proposed model of the mechanism underlying zPER2:zCRY1a-mediated regulation of behavior rhythm and locomotor activity. Left: zPER2, zCRY1a, and zCRY2a synchronize cellular clocks to form zebrafish's behavioral rhythms. Center: the zPER2:zCRY1a complex regulates activity of transcription factor(s) other than the CLOCK (NPAS2):BMAL complex to control cellular metabolism, which is required for maintenance of proper locomotor activity. A potential factor targeted by the zPER2:zCRY1a complex is HIF-1α, which positively regulates genes encoding glycolytic metabolism under normoxic conditions. Right: mouse PER2 physically interacts with nuclear receptors PPARγ to regulate its transcriptional activity.

Supplementary Fig. S13. Uncropped gel images. The images for data of Supplementary Fig. S1 A (A), Supplementary Fig.S5 A (B), Supplementary Fig. S5 B (C) and Supplementary Fig. 5D (D) are shown. Red boxes highlight the cropped segment presented in supplementary figures.

Supplementary Table S1.
List of genes induced in DKO animal treated with 12-h light

Order	Gene name	Order	Gene name
1	hepcidin antimicrobial peptide	21	solute carrier family 25, member 47a
2	zPer2	22	Rh family, C glycoprotein a
3	zCry2b	23	nicotinamide nucleotide adenylyltransferase 1
4	prostaglandin D2 synthase b	24	F-box protein 30b
5	6-4 photolyase	25	proteasome activator subunit 4a
6	phosphodiesterase 6H, cGMP-specific, cone, gamma	26	phosphoenolpyruvate carboxykinase 1 (soluble)
7	cytochrome P450, family 24, subfamily A, polypeptide 1	27	tripartite motif containing 63a
8	LON peptidase N-terminal domain and ring finger 1, like	28	Rh family, C glycoprotein a
9	cartilage acidic protein 1a	29	nuclear factor, interleukin 3 regulated, member 6
10	insulin-like growth factor binding protein 1b	30	family with sequence similarity 134, member B
11	Rho GTPase activating protein 35	31	phosphoenolpyruvate carboxykinase 1 (soluble)
12	tyrosine aminotransferase	32	recoverin 3
13	heme-binding protein soul5	33	kelch-like family member 38b
14	ADP-ribosylation factor 4b	34	pyruvate dehydrogenase kinase, isozyme 2b
15	zCry1a	35	scavenger receptor class B, member 1
16	beta-carotene oxygenase 1, like	36	uncoupling protein 3
17	damage-specific DNA binding protein 2	37	heme oxygenase 1b
18	arrestin 3b, retinal (X-arrestin)	38	uncoupling protein 1
19	ATP-binding cassette, sub-family G (WHITE), member 2a	39	zCry2a
20	arginase 2	40	nuclear factor, interleukin 3 regulated, member 6

Supplementary Table S2. List of genes included in the cluster 6 in Figure 4A

Gene Name	Gene Name
phosducin b	selenophosphate synthetase 1
zgc:162144	heterogeneous nuclear ribonucleoprotein A/Bb
cytochrome P450, family 2, subfamily AD, polypeptide 2	WD repeat and HMG-box DNA binding protein 1
ubiquitin-like with PHD and ring finger domains 1	phosducin a
ligase I, DNA, ATP-dependent	uncharacterized LOC553381
denticleless E3 ubiquitin protein ligase homolog (Drosophila)	si:ch211-132b12.7
origin recognition complex, subunit 1	exonuclease 1
period circadian clock 1b	tonsoku-like, DNA repair protein
si:ch211-203k16.3	minichromosome maintenance complex component 3
crystallin, beta B1	circadian associated repressor of transcription a
transcription factor CP2-like 1	family with sequence similarity 46, member Ab
DNA (cytosine-5-)-methyltransferase 3 beta, duplicate b.2	minichromosome maintenance complex component 2
maternal embryonic leucine zipper kinase	D site albumin promoter binding protein b
ubiquitin-like with PHD and ring finger domains 1	nuclear receptor subfamily 1, group d, member 1
cyclin A2	minichromosome maintenance 10 replication initiation factor
minichromosome maintenance complex component 6	tubulin, beta 2b
adiponectin, C1Q and collagen domain containing, b	3-hydroxy-3-methylglutaryl-CoA reductase a
phosducin b	hes family bHLH transcription factor 6
checkpoint kinase 1	dehydrogenase/reductase (SDR family) member 13 like 1
phosducin b	CTF18, chromosome transmission fidelity factor 18 homolog (S. cerevisiae)
selenophosphate synthetase 1	basic helix-loop-helix family, member e41
solute carrier family 1 (glial high affinity glutamate transporter), member 2a	transglutaminase 2, C polypeptide A
BUB1 mitotic checkpoint serine/threonine kinase Bb	G protein-coupled receptor kinase 7b
si:dkey-285b23.4	phosphatidylinositol 4-kinase, catalytic, alpha b
uncharacterized LOC560010	glycine amidinotransferase (L-arginine:glycine amidinotransferase)
pseudopodium-enriched atypical kinase 1	guanine nucleotide binding protein (G protein), alpha transducing activity polypeptide 2
reticulon 2b	period circadian clock 1a
cyclin E1	echinoderm microtubule associated protein like 1
transferrin receptor 1a	cryptochrome circadian clock 1ab
phosphodiesterase 6C, cGMP-specific, cone, alpha prime	DNA replication and sister chromatid cohesion 1
cyclin E2	X-ray repair complementing defective repair in Chinese hamster cells 4

Supplementary Table S2. (Continued)

Gene Name	Gene Name
establishment of sister chromatid cohesion N-acetyltransferase 2	minichromosome maintenance complex component 2
zgc:162331	cell division cycle 6 homolog (S. cerevisiae)
zgc:153102	guanine nucleotide binding protein (G protein), alpha transducing activity polypeptide 2
muscle-related coiled-coil protein a	minichromosome maintenance complex component 4
regulator of chromosome condensation 1	minichromosome maintenance complex component 5
CTF18, chromosome transmission fidelity factor 18 homolog (S. cerevisiae)	G protein-coupled receptor kinase 7b
si:dkey-57a22.11	cyclin B1
acyl-CoA synthetase family member 2	5-methyltetrahydrofolate-homocysteine methyltransferase
prolactin receptor a	hyaluronan-mediated motility receptor (RHAMM)
si:ch211-132b12.7	cryptochrome circadian clock 2
nuclear receptor subfamily 1, group d, member 1	selenophosphate synthetase 1
tonsoku-like, DNA repair protein	ligase I, DNA, ATP-dependent
chondroitin sulfate N-acetylgalactosaminyltransferase 1	cell division cycle associated 7a
zinc finger protein 395b	family with sequence similarity 183, member A
minichromosome maintenance complex component 5	cerebellin 14
calcium/calmodulin-dependent protein kinase kinase 1, alpha b	transmembrane protein 206
UDP-glucose 6-dehydrogenase	helicase, lymphoid-specific
transcription factor CP2-like 1	minichromosome maintenance complex component 5
acyl-CoA synthetase family member 2	tonsoku-like, DNA repair protein
alpha-2-HS-glycoprotein 2	minichromosome maintenance complex component 4
alpha-2-HS-glycoprotein 2	CLOCK-interacting pacemaker-like
solute carrier family 1 (glutamate transporter), member 8b	ligase I, DNA, ATP-dependent
cyclin A2	minichromosome maintenance complex component 5
potassium voltage-gated channel, Shab-related subfamily, member 2	acyl-CoA synthetase short-chain family member 2 like
minichromosome maintenance complex component 6	checkpoint kinase 2
polymerase (DNA directed), alpha 2	3-hydroxy-3-methylglutaryl-CoA reductase a
helicase, lymphoid-specific	period circadian clock 3
LIM domain kinase 1a	minichromosome maintenance complex component 4
guanine nucleotide binding protein (G protein), alpha transducing activity polypeptide 2	neurocalcin delta b
synaptopodin 2b	si:ch211-132b12.2
hyaluronan-mediated motility receptor (RHAMM)	caspase b
circadian associated repressor of transcription a	si:ch211-132b12.1

Supplementary Table S3. Results of cosinor analysis

			P value
WT vs DKO 3h Light	***zPer1***	WT	0.0003
		DKO	0.9861
	zRev-erb α	WT	0.0001
		DKO	0.8663
	zPer3	WT	0.0145
		DKO	0.9565
WT vs DKO 12h Light	***zPer1***	WT	0.0001
		DKO	0.0001
	zRev-erb α	WT	0.0020
		DKO	0.0282
	zPer3	WT	0.0003
		DKO	0.0029
WT vs TKO 12h Light	***zPer1***	WT	0.0122
		TKO	0.9938
	zRev-erb α	WT	0.0739
		TKO	1.0000
	zPer3	WT	0.0015
		TKO	0.9948

Supplementary Table S4. Results of analysis by Two Way ANOVA´s method

		P value
WT vs DKO 3h Light	*zPer1*	1.1E-08
	zRev-erbα	0.1146
	zPer3	1.6E-07
WT vs DKO 12h Light	*zPer1*	9.2E-07
	zRev-erbα	0.8923
	zPer3	0.6314
WT vs TKO 12h Light	*zPer1*	0.0797
	zRev-erbα	0.1676
	zPer3	0.662

Supplementary Table S5.
Summary of phenotypes of WT, DKO and TKO animals

	**Wild type**	***zPer2*$^{-/-}$ *zCry1a*$^{-/-}$**	***zPer2*$^{-/-}$ *zCry1a*$^{-/-}$ *zCry2a*$^{-/-}$**
3 h light			
Locomotor activity	Normal	Impaired	Impaired
Behavior rhythm	Formed	Not formed	Not formed
Circadian rhythm in gene expression	Formed	Not formed	Not formed
12 h light			
Locomotor activity	Normal	Impaired	Impaired
Behavior rhythm	Formed	Formed	Not formed
Circadian rhythm in gene expression	Formed	Formed	Not formed

Dash lines are the borderlines which separate normal phenotypes from abnormal ones.

Supplementary Table S6. Primer sequences used for quantitative RT-PCR

Target gene	Forward sequence	Reverse sequence
zPer1	5'-CCGTCAGTTTCGCTTTTCTC-3'	5'-ATGTGCAGGCTGTAGATCCC-3'
zPer2	5'-ATGTCGATGGCTTTAGGCAG-3'	5'-CGAGACATCCAGAAGGTGCT-3'
zPer3	5'-CCTTCGGAAACAAGCCATTA-3'	5'-CCGCCTCCATAGAAAATCAA-3'
zCry1a	5'-TCCGCTGTGTGTACATCCTC-3'	5'-CAAACACTGCAGCAAAAACC-3
zCry1b	5'-AACAGCTTGTCAACAGTGTG CAG-3'	5'-AATCTGTTGTTCGGTCTTCA CCA-3'
zCry2a	5'-ACATGCCTGACTCGGGCCACAA CAG-3'	5'-CTAGATCTCGTTCCGACTCTCTC TC-3'
zCry2b	5'-CCACGACTCTTCAAGGAATGGA-3'	5'-CACGCTAGTTTCTTTATCGCCG-3'
zCry3	5'-AGCCTCCCATGTCAGATGACT CT-3'	5'-GATTCAGACAGCGGTGGTGTA GA-3'
zCry4	5'-GTAAAGACTATCCCCGGCCAA TA-3'	5'-CATCGGGTCATCTGCTACATC TC-3'
zCry -Dash	5'-CACAGGTCTCACTCAACCAAA CA-3'	5'-CCCTTTCTTCCTTTAGATGATG AC-3'
zRev -erba	5'-GGACAAGCCAGCAGAATCTC-3'	5'-CCTGAAAAACATCAGCAGCA-3'
zAanat2	5'-GTGTCCTGAGCTGTCCATGGGC TGG-3'	5'-GAGTCCGGCACGTGTGTGCTCA TAG-3'
zActin	5'-GCAGATGTGGATCAGCAAGCA GG-3'	5'-CTGAGTCAATGCGCCATACAG AG-3'

A

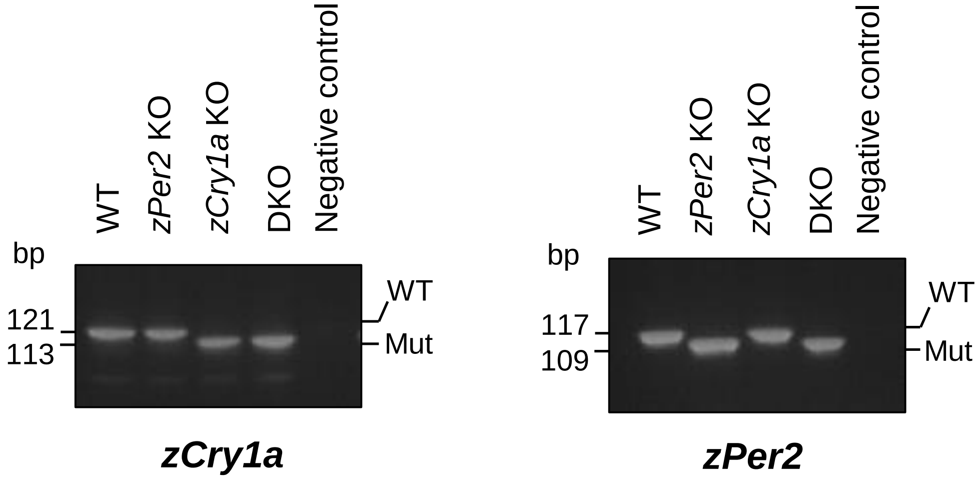

B

zCry1a

WT 5'-TCCACGACAATCCTTcactcagagactctaTTCTGGGAGCGCAC-3'
Mut 5'-TCCACGACAATCCTTcact--------ctaTTCTGGGAGCGCAC-3'

zPer2

WT 5'-TCATTCCCTACATGAggatgtggagatgaagAGTAGCAGCGGGTCCA-3'
Mut 5'-TCATTCCCTACATGAggat--------gaagAGTAGCAGCGGGTCCA-3'

Supplementary Fig. S1

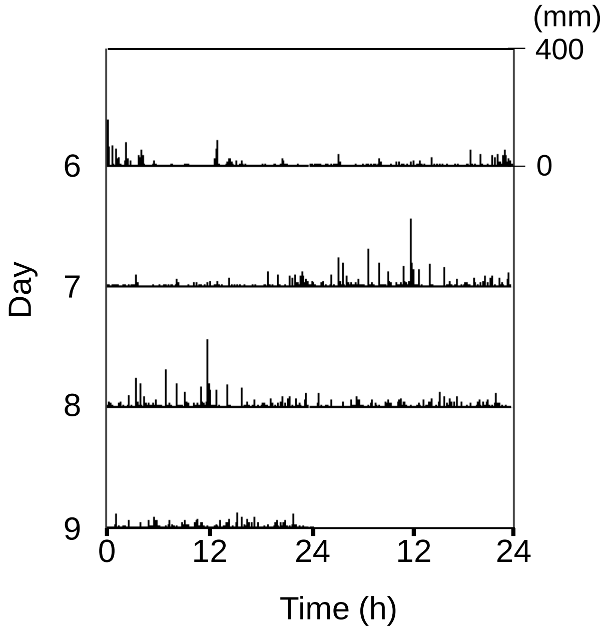

Supplementary Fig. S2

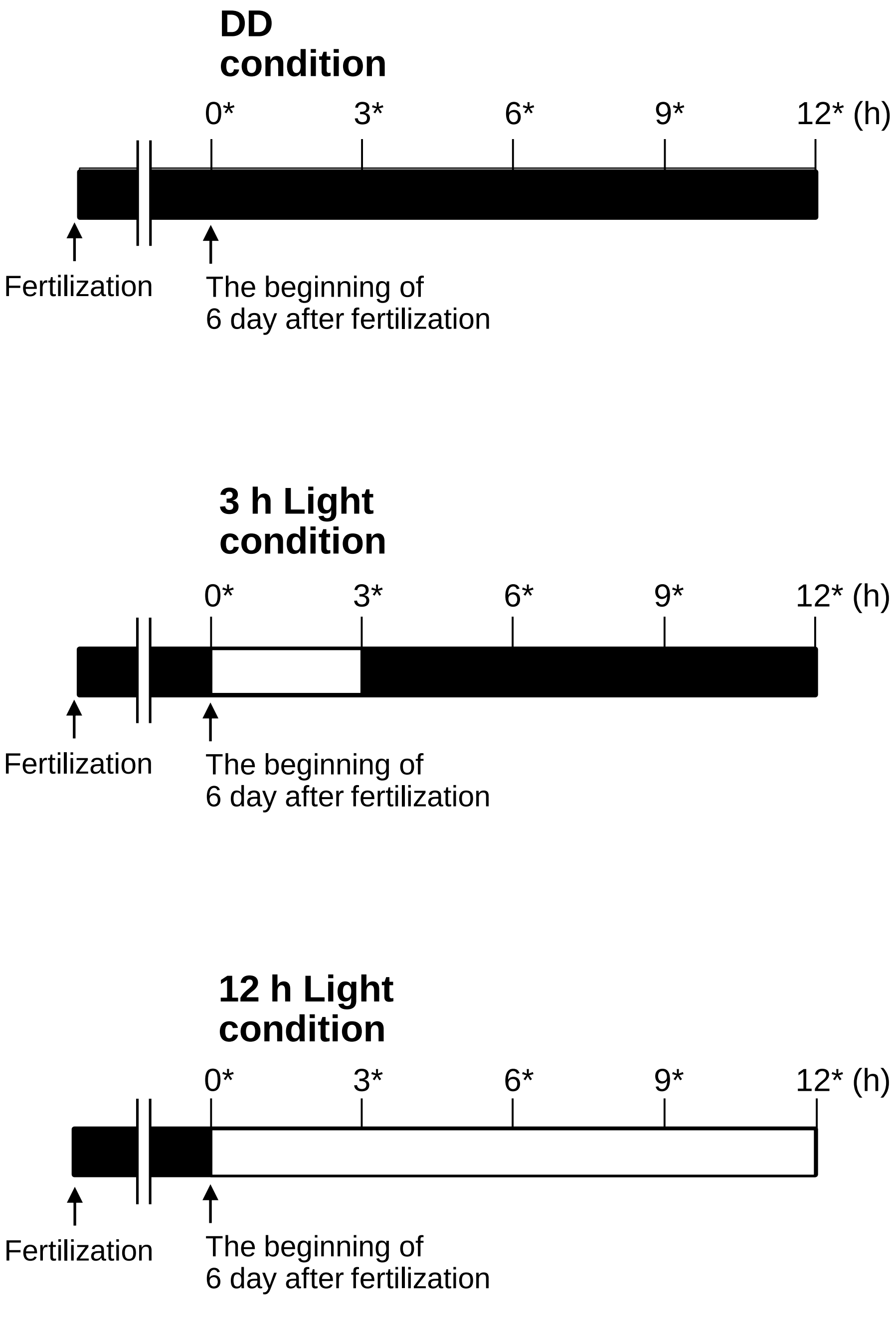

Supplementary Fig. S3

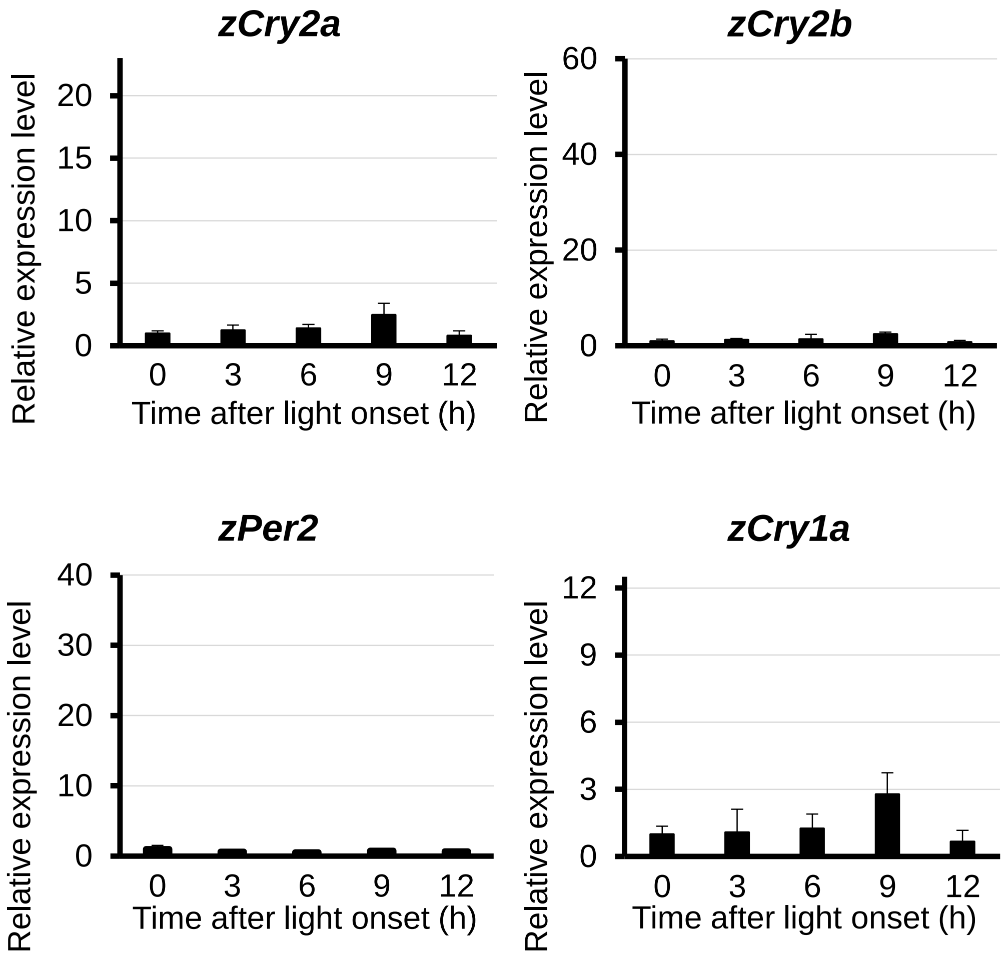

Supplementary Fig. S4

A

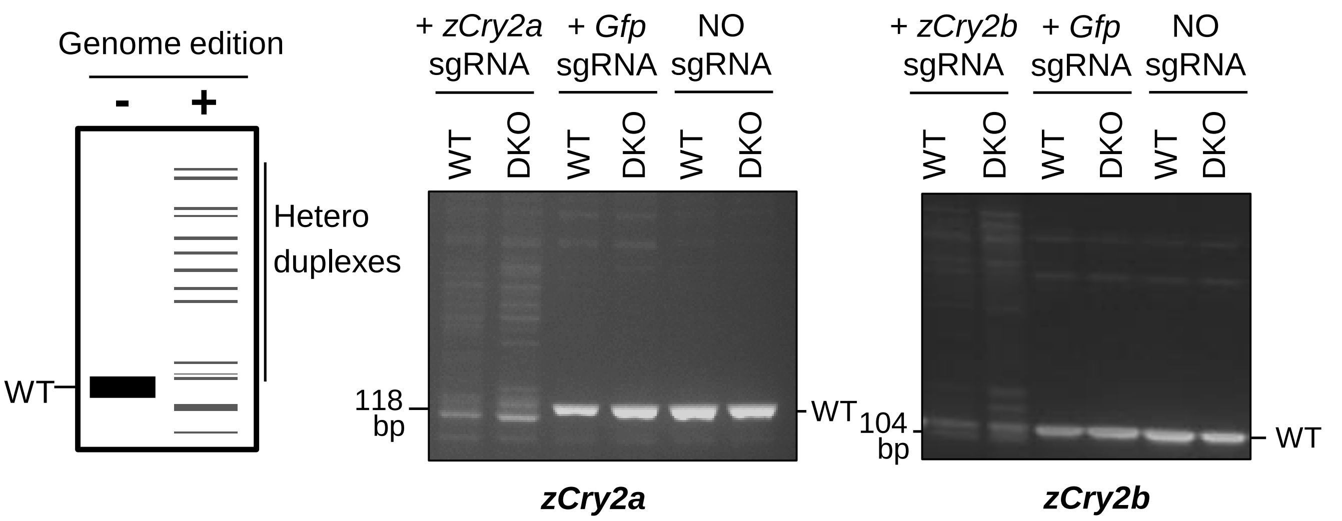

B

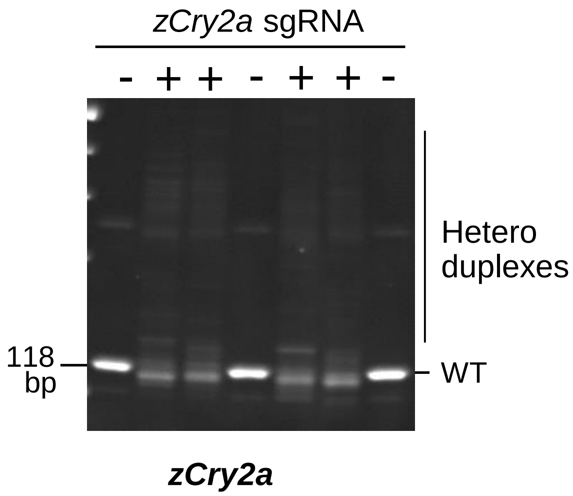

C

zCry2a WT

5’-ATGACTCGGaaccgtttgggaaggaga<u>GGG</u>ATG-3’

zCry2a Mut

5’-ATGACTCGGaaccgtttgggaa- - - - -GGGATG-3’

D

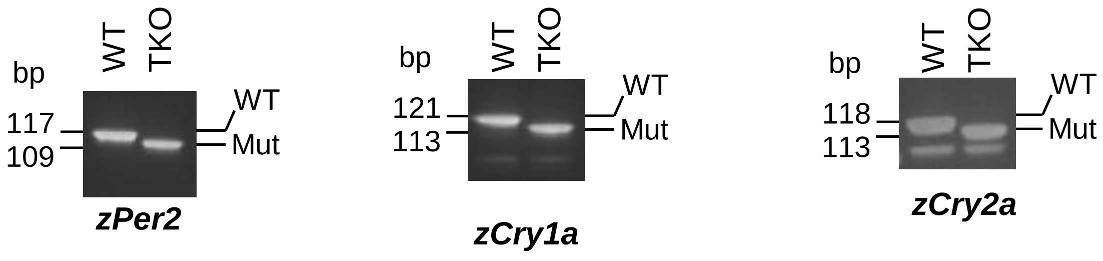

Supplementary Fig. S5

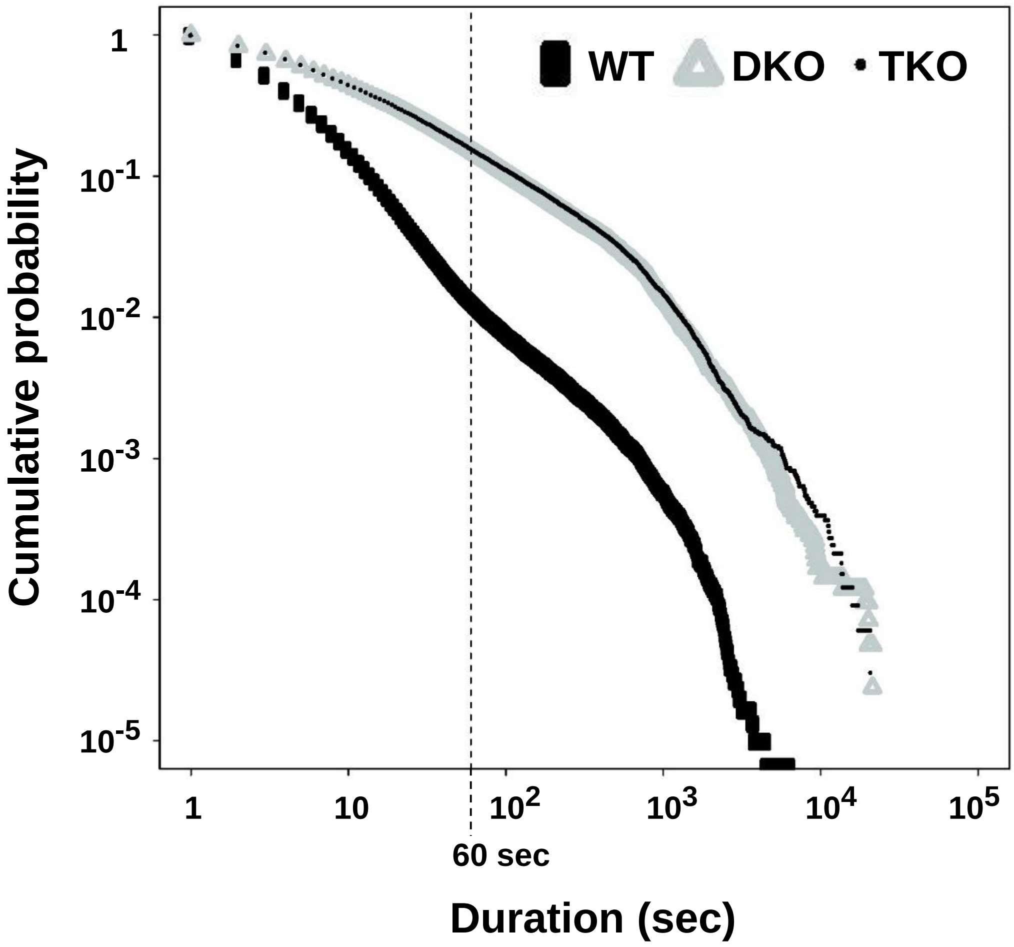

Supplementary Fig. S6

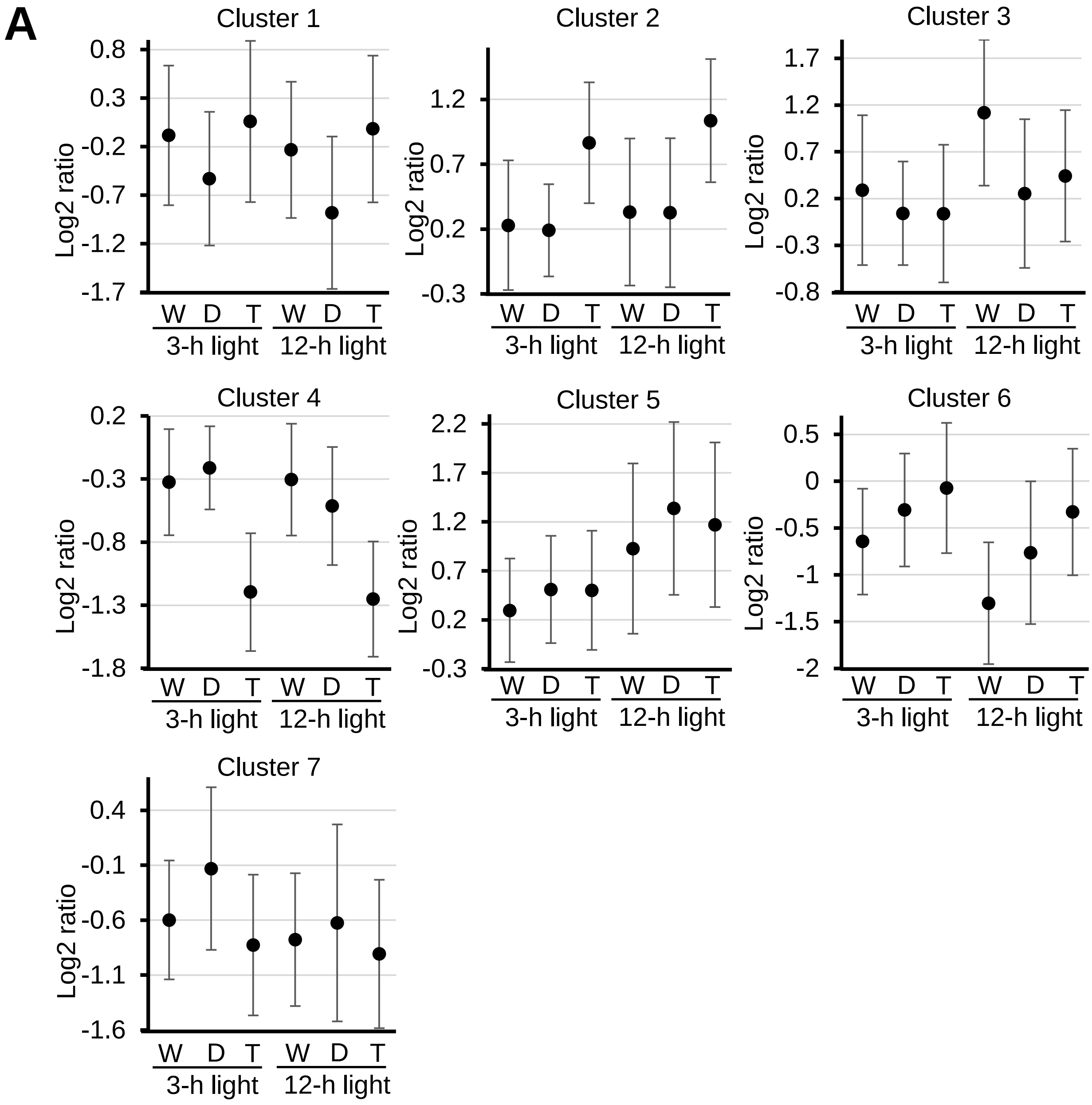

B

Pathway	Score (p value)
RB/E2F-dependent transcription	6.7E-12
ATM/ATR	1.6E-11
CLOCK:BMAL-dependent transcription	2.0E-11
14-3-3 signaling	1.8E-10
Inflammasome signaling	9.0E-09

Pathway	Score (p value)
Caspase signaling	5.3E-06
Transcriptional regulation by FOXM	3.3E-05
Adipokine signaling	9.7E-05
CYP family	1.1E-04
Bombesin signaling	3.1E-04

Supplementary Fig. S7

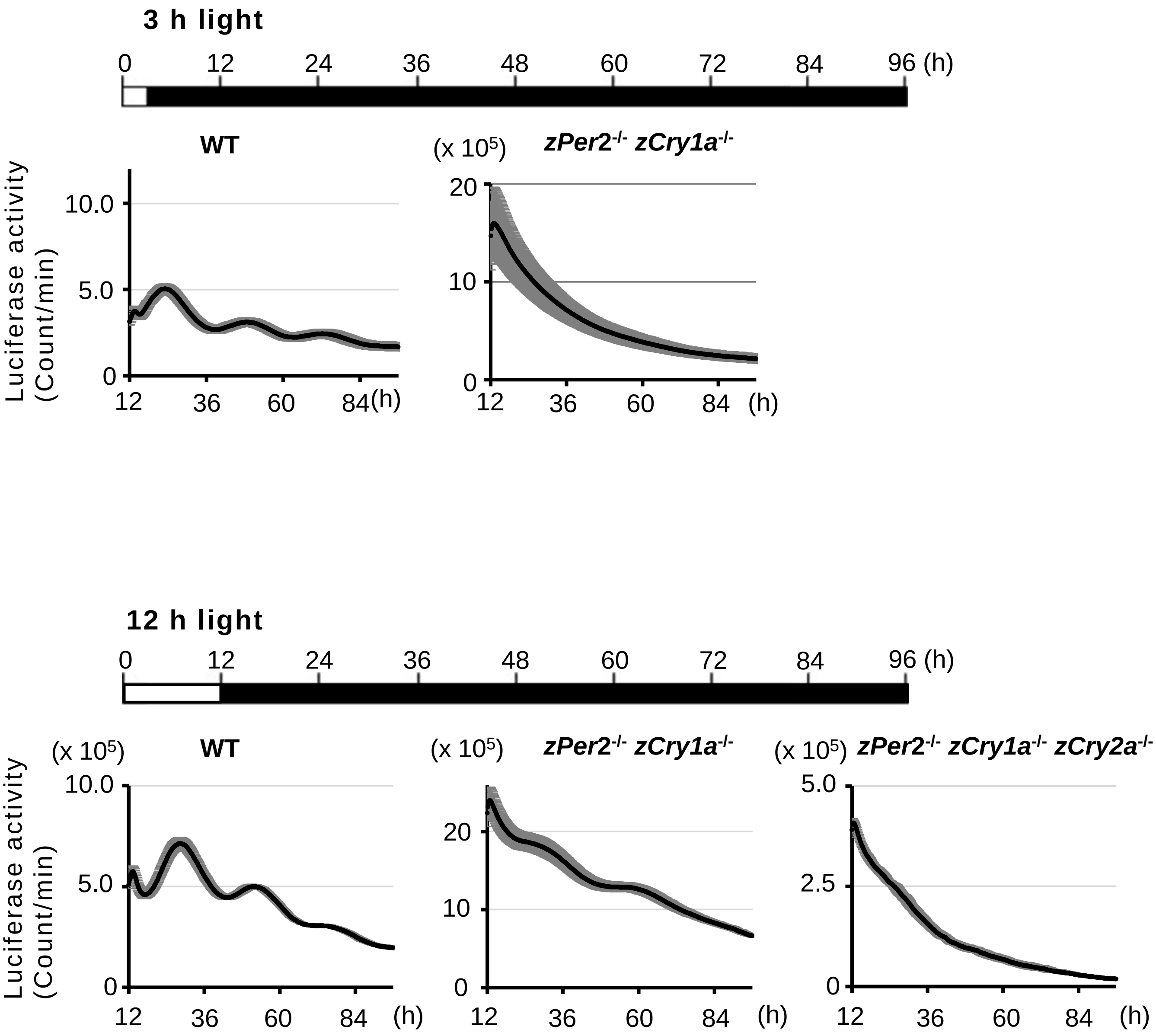

Supplementary Fig. S8

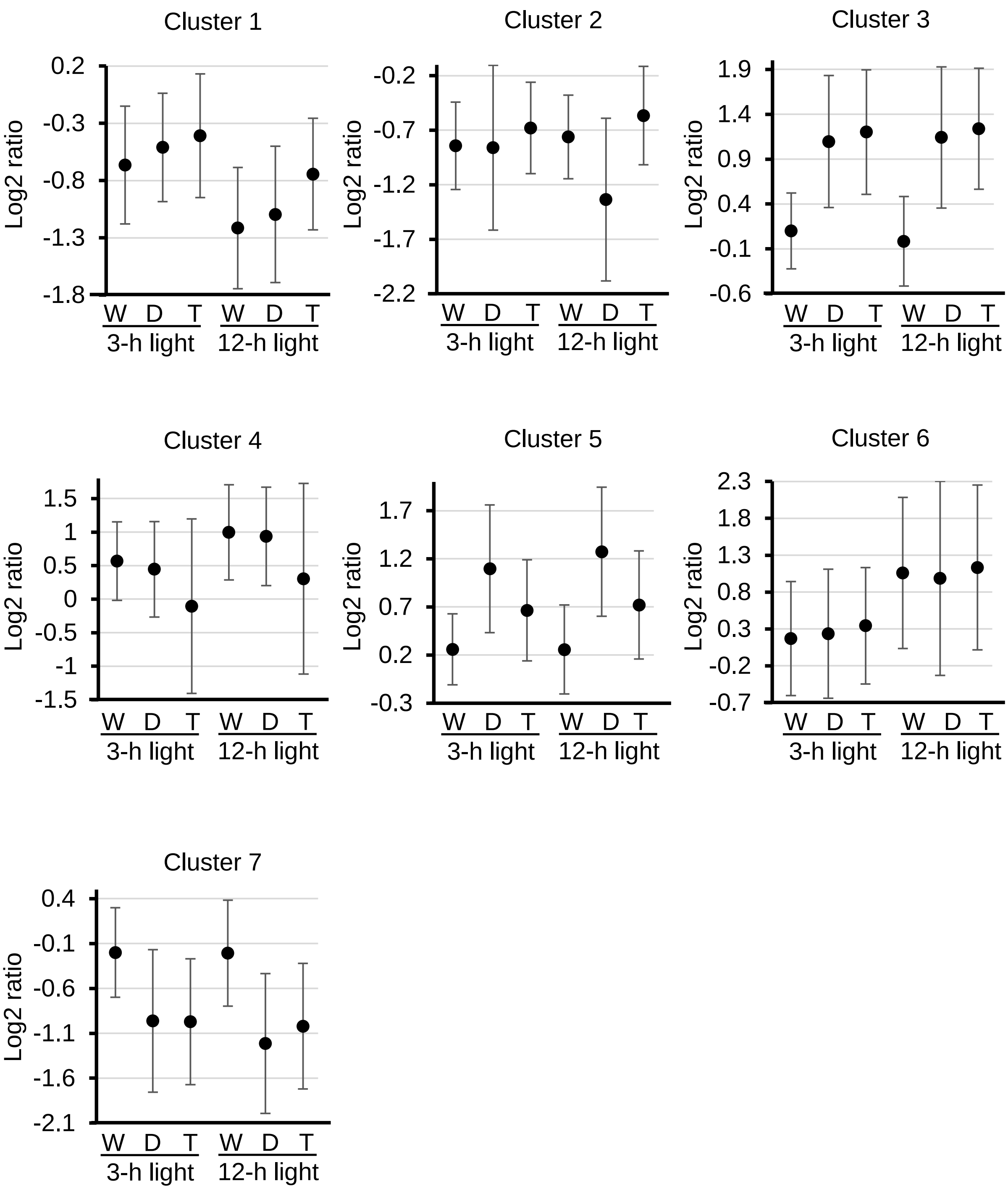

Supplementary Fig. S9

1. The cluster analysis of microarray data and GO analysis of each identified 7 clusters.
(7 clusters were identified, and the top 6 GO terms for each cluster were shown in Fig S11)

↓ Filtration by clusters of genes whose expression was commonly changed in DKO and TKO animals, but not in WT animal.

2. GO terms in clusters 3 and 7 were selected.

↓ Search the GO terms related to the animal’s locomotor activity

3. GO terms related to metabolism were selected.

↓ Genes constituting the GO terms were analyzed.

4. Genes regulating cellular ATP levels were selected.

Supplementary Fig. S10

Cluster 1's GO	Score (p value)
DNA-dependent DNA replication	1.11E-24
DNA replication	4.41E-24
DNA strand elongation involved in DNA replication	3.16E-22
DNA strand elongation	1.71E-21
DNA metabolic process	1.37E-19
Response to abiotic stimulus	2.89E-19

Cluster 2's GO	Score (p value)
Protein complex assembly	1.73E-32
Mitotic cell cycle	1.90E-30
Cell cycle	4.59E-29
Protein-DNA complex subunit organization	7.58E-29
DNA conformation change	8.73E-28
Nucleosome assembly	7.63E-27

Cluster 3's GO	Score (p value)
Single-organism process	1.71E-31
Metabolic process	1.80E-23
Cellular process	1.92E-23
Single-organism cellular process	2.06E-23
Regulation of biological quality	1.10E-22
Single-organism metabolic process	2.36E-22

Cluster 4's GO	Score (p value)
Complement activation alternative pathway	1.02E-29
Positive regulation of apoptotic cell clearance	7.09E-28
Positive regulation of GPCR signaling pathway	345E-26
Regulation of triglyceride bio-synthetic process	6.05E-26
Regulation of apoptotic cell clearance	7.61E-25
Positive regulation of lipid storage	1.68E-24

Cluster 5's GO	Score (p value)
Cellular heat acclimation	3.36E-14
Heart acclimation	3.36E-14
Positive regulation of endoribonuclease activity	4.88E-13
Positive regulation of mRNA cleavage	4.88E-13
Regulation of mRNA cleavage	3.39E-12
Negative regulation of intracellular signal transduction	6.08E-11

Cluster 6's GO	Score (p value)
Detection of stimulus	4.91E-25
Response to radiation	5.20E-23
Response to light stimulus	3.62E-21
Phototrasduction	6.24E-21
Response to abiotic stimulus	1.13E-20
Phototrasduction, visible light	1.40E-20

Cluster 7's GO	Score (p value)
Regulation of biological quality	7.43E-45
Oxidation-reduction process	2.22E-38
Single-organism metabolic process	2.94E-37
Response to chemical	1.09E-35
Neural nucleus development	2.70E-35
Substantia nigra development	3.09E-35

Supplementary Fig. S11

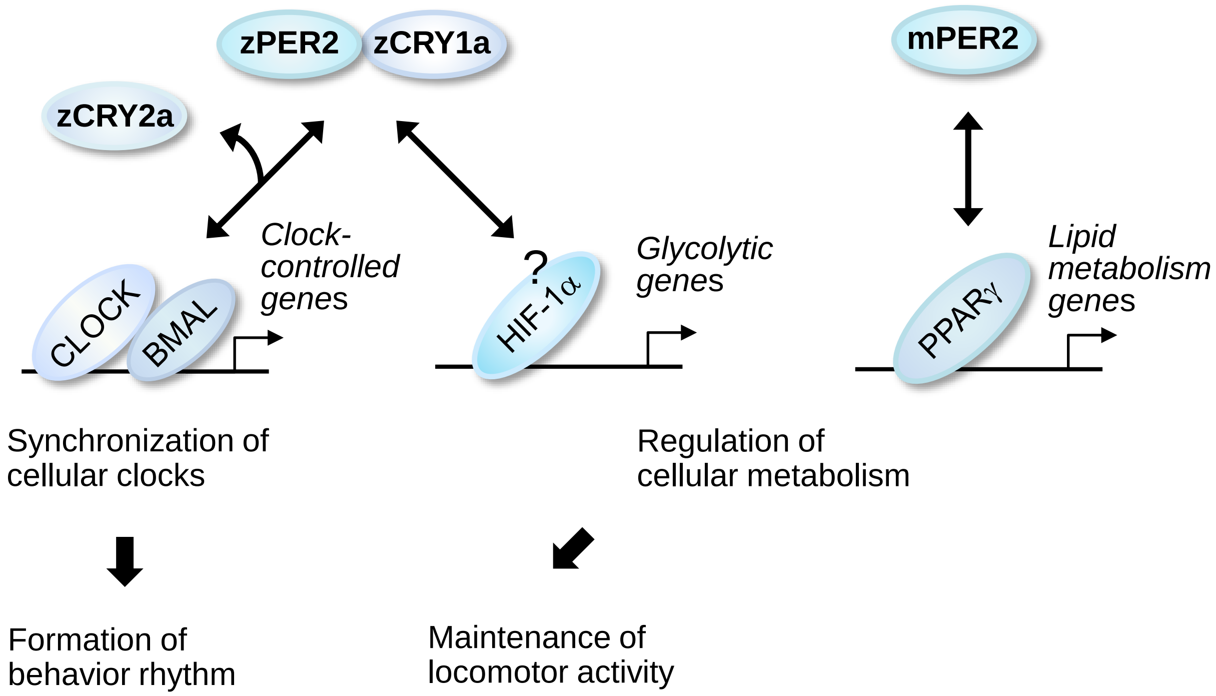

Supplementary Fig. S12

A

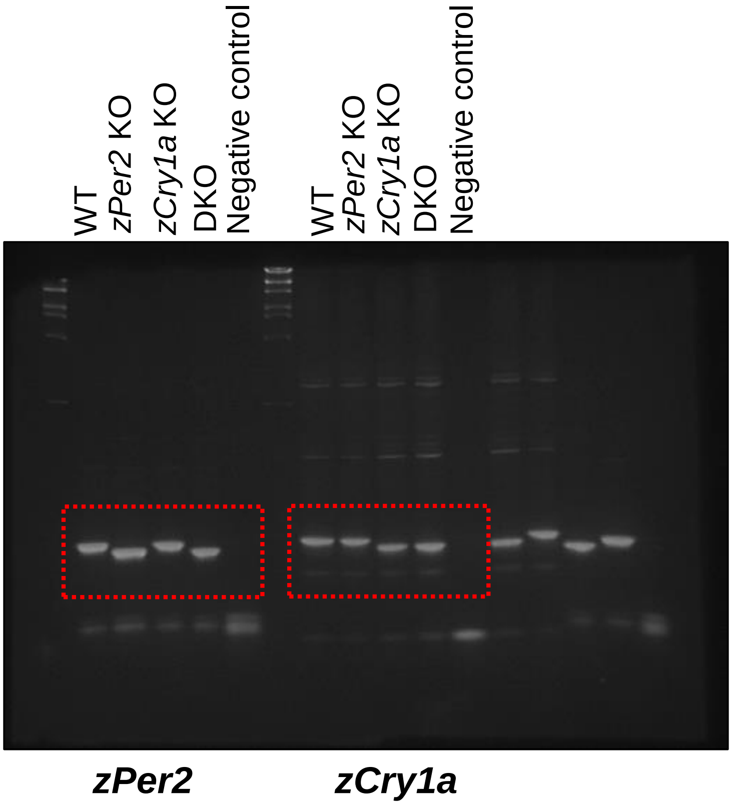

Supplementary Fig. S13

B

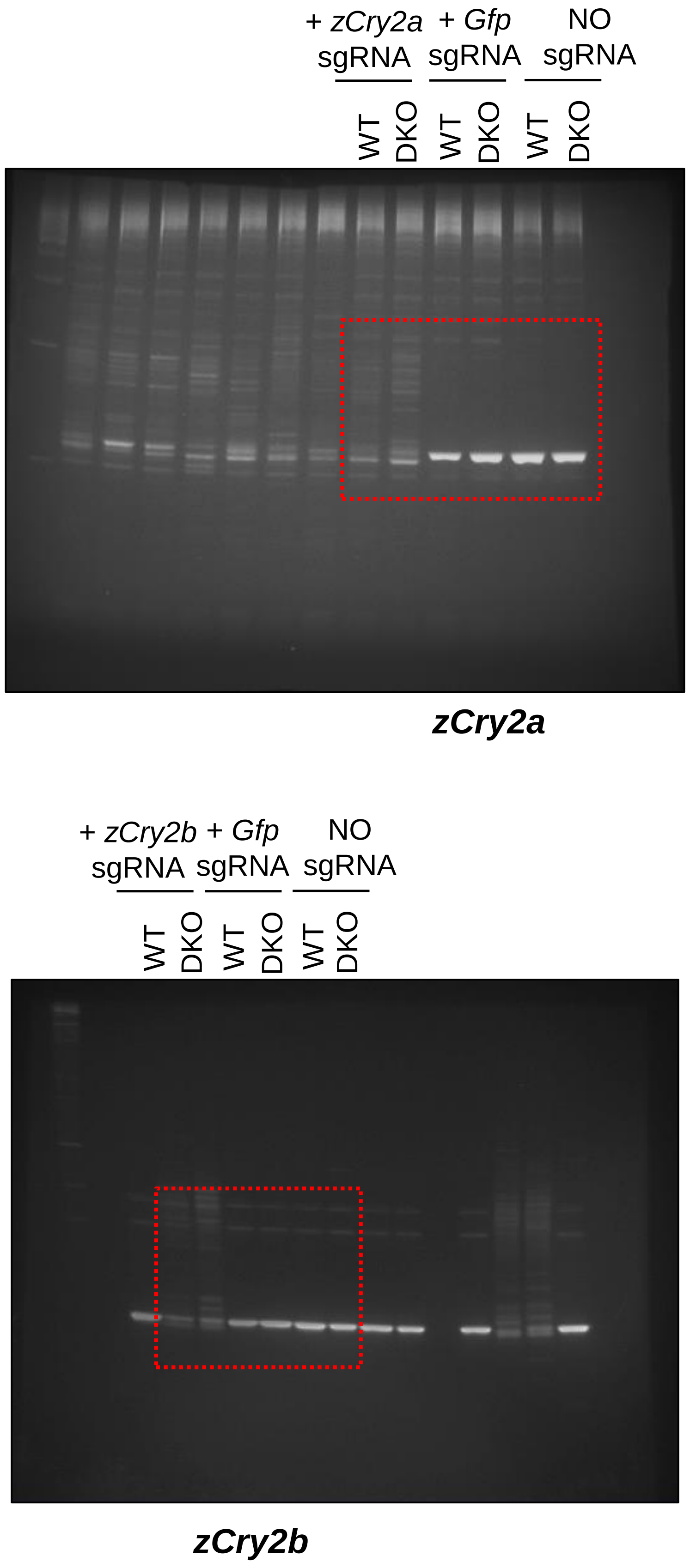

Supplementary Fig. S13

C

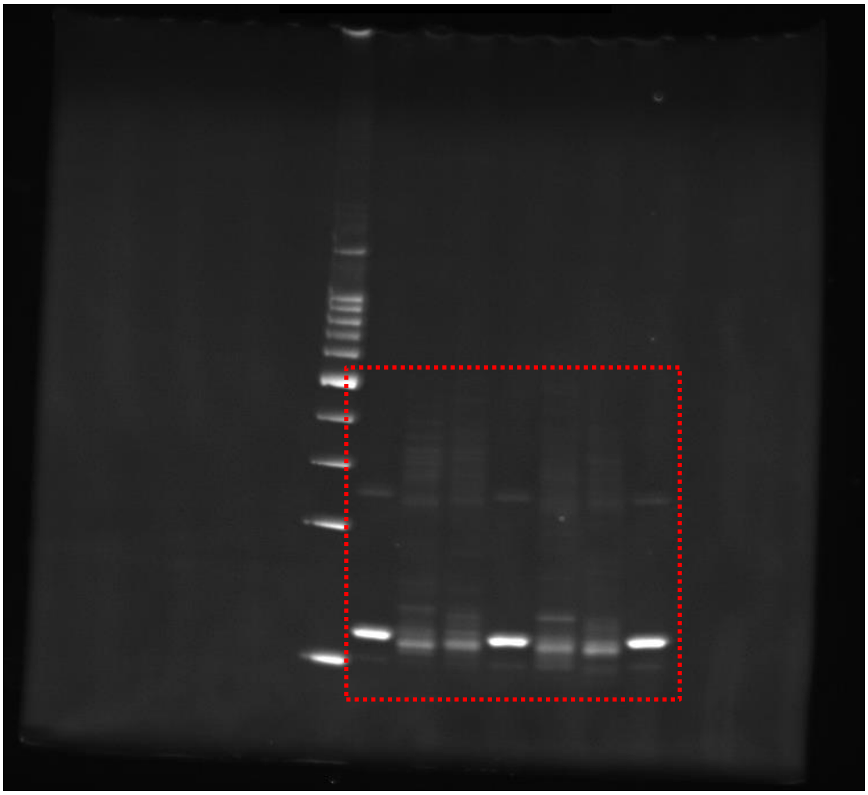

Supplementary Fig. S13

D

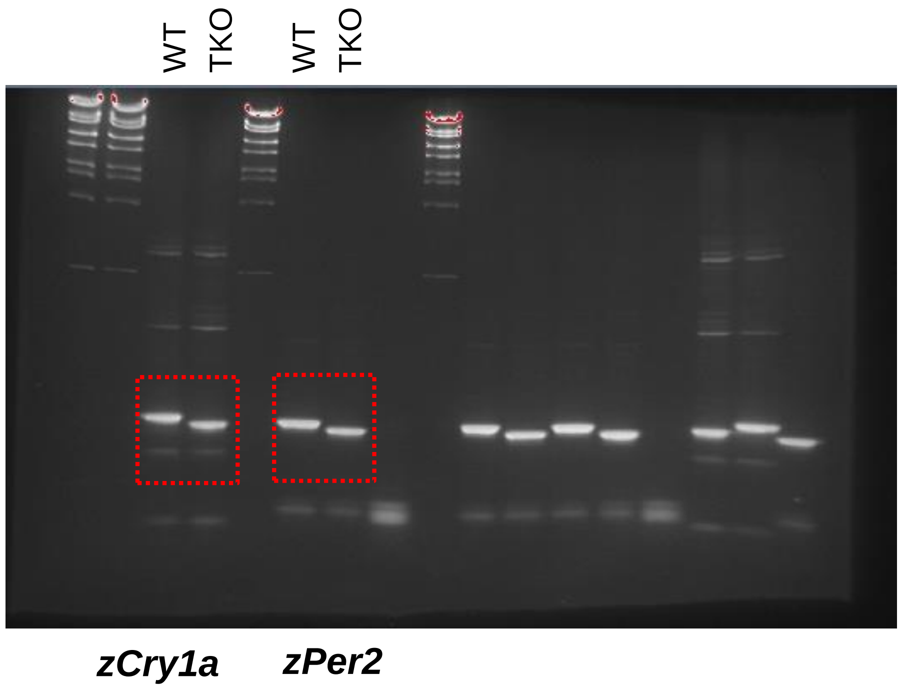

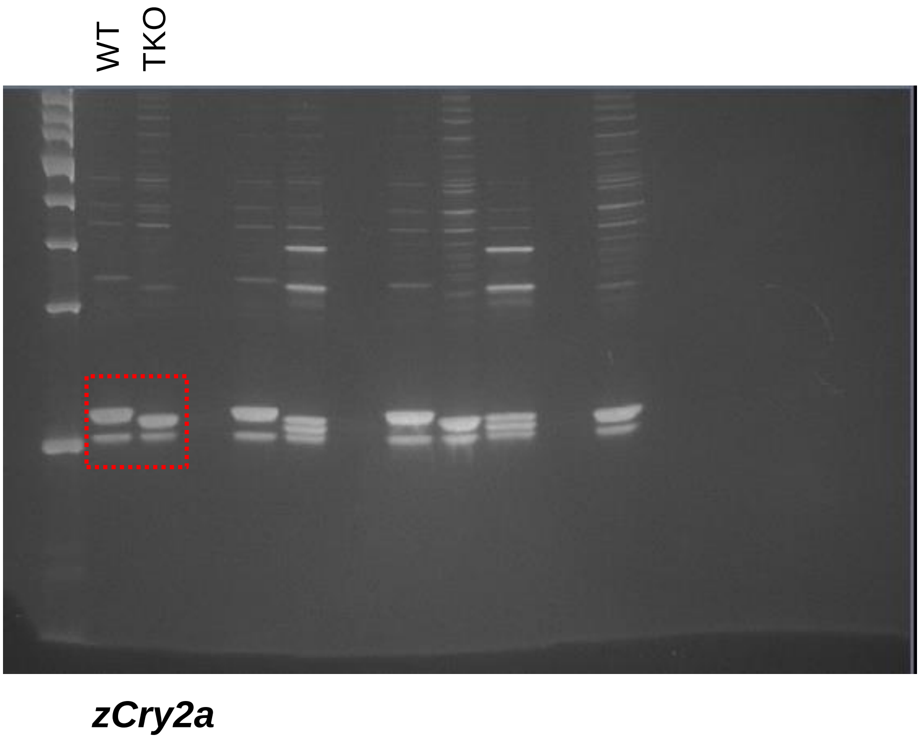

Supplementary Fig. S13
